# Genome-wide characterization of the GRF transcription factors in potato (*Solanum tuberosum* L.) and expression analysis of *StGRF* genes during potato tuber dormancy and sprouting

**DOI:** 10.3389/fpls.2024.1417204

**Published:** 2024-06-24

**Authors:** Danni Cui, Yin Song, Weihao Jiang, Han Ye, Shipeng Wang, Li Yuan, Bailin Liu

**Affiliations:** ^1^ Shenzhen Research Institute, Northwest A&F University, Shenzhen, China; ^2^ State Key Laboratory for Crop Stress Resistance and High-Efficiency Production, College of Agronomy, Northwest A&F University, Yangling, China

**Keywords:** GRF transcription factors, gene expression, regulatory network, tuber dormancy, sprouting, potato

## Abstract

Growth-regulating factors (GRFs) are transcription factors that play a pivotal role in plant growth and development. This study identifies 12 *Solanum tuberosum* GRF transcription factors (StGRFs) and analyzes their physicochemical properties, phylogenetic relationships, gene structures and gene expression patterns using bioinformatics. The StGRFs exhibit a length range of 266 to 599 amino acids, with a molecular weight of 26.02 to 64.52 kDa. The majority of *StGRFs* possess three introns. The promoter regions contain a plethora of *cis*-acting elements related to plant growth and development, as well as environmental stress and hormone response. All the members of the StGRF family contain conserved WRC and QLQ domains, with the sequences of these two conserved domain modules exhibiting high levels of conservation. Transcriptomic data indicates that *StGRFs* play a significant role in the growth and development of stamens, roots, young tubers, and other tissues or organs in potatoes. Furthermore, a few *StGRFs* exhibit differential expression patterns in response to *Phytophthora infestans*, chemical elicitors, heat, salt, and drought stresses, as well as multiple hormone treatments. The results of the expression analysis indicate that *StGRF1*, *StGRF2*, *StGRF5*, *StGRF7*, *StGRF10* and *StGRF12* are involved in the process of tuber sprouting, while *StGRF4* and *StGRF9* may play a role in tuber dormancy. These findings offer valuable insights that can be used to investigate the roles of *StGRFs* during potato tuber dormancy and sprouting.

## Introduction

Transcription factors (TFs), also referred to as *trans*-acting elements, can bind to specific sequences (*cis*-acting elements) in the gene promoter region to regulate the expression of a target gene. TFs exhibit a wide range of functions and play crucial roles in numerous biological processes and regulatory pathways in plants. To date, 320,370 transcription factors have been identified and classified into 58 families across 165 species (Jin et al., 2017). Among these families, growth-regulating factors (GRFs) are a plant-specific type of TF that were originally identified for their roles in stem and leaf development in rice (van der Knaap et al., 2000). To date, members of the GRF family have been identified in a number of plant species, including thale cress (*Arabidopsis thaliana*), maize (*Zea mays*), rice (*Oryza sativa*), oilseed rape (*Brassica napus*), soybean (*Glycine max*), tomato (*Solanum lycopersicum*), and moss (*Physcomitrella patens*) (Kim et al., 2003; Choi et al., 2004; Zhang et al., 2008; Khatun et al., 2017; Ma et al., 2017; Chen et al., 2019). The GRF proteins share common features, including the QLQ (Gln, Leu, Gln) and WRC (Trp, Arg, Cys) domains at the N-terminus, and the relatively variable regions at the C-terminus. They form complexes with their co-activators, known as GRF-interacting factors (GIFs), which can bind to the *cis*-acting region of downstream target genes and regulate their expression, inferring a role in transcriptional regulation.

GRF family genes are involved in the growth, development, and regeneration of plants. In *Arabidopsis*, *AtGRF1*, *AtGRF2*, and *AtGRF3* are predominantly expressed in shoots, roots, and stems (Kim et al., 2003). *AtGRF4* and *AtGRF6* are expressed in the midvein of leaves, while *AtGRF5* is expressed in leaf primordia (Horiguchi et al., 2005). *AtGRF7* is expressed in the blades and petioles of true leaves, while *AtGRF7* and *AtGRF8* are predominantly expressed in the shoot tips (Kim et al., 2003). Additionally, *AtGRF1*, *AtGRF2*, *AtGRF4*, *AtGRF5*, *AtGRF6*, *AtGRF7*, and *AtGRF9* are expressed in the flower (http://bar.utoronto.ca/eplant/). *AtGRF1*, *AtGRF2*, *AtGRF3*, *AtGRF4*, *AtGRF5*, and *AtGRF7* are strongly expressed in the meristematic zone (Kim et al., 2003; Rodriguez et al., 2010; Kim et al., 2012; Bao et al., 2014 **;** **Pajoro et al., 2014**). GRFs have been reported to alter leaf cell numbers, thereby affecting the leaf size and longevity (Debernardi et al., 2014 **;** **Wu et al., 2014**; Nelissen et al., 2015; Vercruyssen et al., 2015), stem elongation (van der Knaap et al., 2000; Kuijt et al., 2014), root development (Hewezi et al., 2012; Bao et al., 2014), floral organ development and regulation of flowering time (Pajoro et al., 2014), and seed oil content (Liu et al., 2012). GIFs act as transcriptional co-regulators of GRFs. GIF1 interacts with AtGRF1, AtGRF2, AtGRF4, AtGRF5 and AtGRF9 through its conserved QLQ domain (Kim and Kende, 2004; Horiguchi et al., 2005). The overexpression of *GIF1*/*AN3* leads to an increase in leaf area due to an increase in cell number (Horiguchi et al., 2005). Conversely, a moderate reduction in *GIF1* expression results in smaller leaves due to a reduction in cell number. Moreover, a number of *GRF* genes contain the miRNA396 target site, and it is known that miRNA396 plays a role in regulating *GRF* genes during plant development (Rodriguez et al., 2010; Debernardi et al., 2012). In *Arabidopsis*, miR396a and miR396b regulate leaf growth and development by post-transcriptional repression of *GRF* genes (Rodriguez et al., 2010; Wang et al., 2011). OsmiR396d targets *OsGRF6* and *OsGRF10*, and overexpression of OsmiR396 results in an abnormal number of stigmas and stamens (Liu et al., 2014). Furthermore, evidences indicate that GRFs play a role in the plant adaptation to stress (Kim et al., 2012; Casadevall et al., 2013 **;** Liu et al., 2014), and are closely associated with plant hormones (Choi et al., 2004; Bazin et al., 2013).

Potato (*Solanum tuberosum* L.) is the third most important food crop worldwide, after rice and wheat. It plays a vital role in ensuring global food security, particularly in light of the growing population and the concomitant increase in hunger. It is estimated that over one billion people worldwide consume potatoes, with global production exceeding 300 million metric tons. The release of the potato’s complete genome sequence has enabled a comprehensive analysis of the *GRF* genes (Xu et al., 2011). GRFs are involved in regulating the growth of different plant tissues and organs. The potato tuber is a swollen underground stem formed by shortened internodes and nodes that develop into tuber eyes. Meristematic activity in the tuber eyes is completely suppressed during the development of the tuber. Even when placed in optimal conditions for sprouting, such as warm temperature, darkness, and high humidity, the tuber buds are generally dormant and will not sprout or grow. Subsequently, the tuber enters a period of dormancy, during which the eye exhibits visible growth of a bud. As tuber dormancy is an important agronomic trait, a short dormancy period renders potato tubers challenging to store for an extended period, whereas a long dormancy period makes them difficult to plant in a timely manner. Tuber sprouting typically originates from the tuber apical meristem (TAM), although GRFs are among the most crucial regulators of meristem development and cell differentiation restriction in the shoot apical meristem (SAM). Nevertheless, the role of GRF genes in potato tuber dormancy and sprouting remains elusive. In order to gain a better understanding of the role of GRF transcription factors in potato, a genome-wide identification and analysis of the potato (*S. tuberosum*) *GRF* family members (*StGRFs*) was conducted. In the present study, a total of 12 *GRF* genes in the potato genome were identified. The expression patterns of *StGRFs* in different tissues were analyzed. Furthermore, the expression profiles of *StGRFs* under multiple external stimuli and hormones, as well as during tuber dormancy were examined. The results demonstrated that *StGRF* genes exhibited distinct expression patterns in different tissues, and that their transcription was induced by diverse biotic and abiotic stresses. Further analysis of the expression patterns of these *StGRFs* revealed that they may play a significant role in regulating the release of tuber dormancy.

## Materials and methods

### Plant materials

A diploid potato line EB063 derived from a cross between parent E (ED25) and B (CW2–1) (Xiao et al., 2018) was utilized in the present study. The sprouted seed tubers were planted at the Yulin potato breeding station, located in the Shaanxi Province of China. Local cultural practices were employed to ensure optimal plant growth. The tubers were harvested at the appropriate stage of maturity and stored in the dark at room temperature for a period of two weeks, during which time they underwent wound healing. Subsequently, tubers of a similar size were then placed in light-proof boxes at a temperature of 22 ± 2°C in order to facilitate the release of dormancy. The tubers were designated as dormant tubers (DT) at the point at which they were fully mature (0 day at dormancy release array), while any sprouts that were ≥2 mm in length were considered to be sprouting tubers (ST). For the purpose of sample collection, the apical bud meristems (also known as the dormancy eye) were excised at day 0 of the dormancy release array. The sprouting sprouts and sprouting sprout bases were collected at five weeks of the dormancy release array, respectively, from three to five tubers using a 6 mm size cork borer. The samples were immediately frozen in liquid nitrogen and stored at −80°C, after which they were subjected to RNA isolation.

### Identification and physicochemical properties analysis of the GRF family members in potato

The potato genome files (version number DM v6.1) were downloaded from the Spud DB database (http://spuddb.uga.edu/). Amino acid sequences of *Arabidopsis* (TAIR, http://www.arabidopsis.org/) and rice (RGAP, http://rice.uga.edu/) GRF proteins were employed as queries in a BlastP homology search to identify candidate GRF proteins against the potato genome DM v6.1. All retrieved amino acid sequences of potato were subjected to verification for the presence of both the QLQ and WRC (PF08880, PF08879, respectively; http://pfam.xfam.org/) domains through the CDD (Conserved Domains Database; https://www.ncbi.nlm.nih.gov/cdd/) and SMART (http://smart.embl-heidelberg.de/) programs. The physiochemical properties of StGRF proteins including the amino acid number, molecular weight (MW), isoelectric point (pI), and grand average of hydropathicity (GRAVY), were analyzed by the ExPASy ProtParam tool (http://www.expasy.org/protparam/). The subcellular localization of StGRF proteins was determined by web-server predictors in the Cell-PLoc package (http://www.csbio.sjtu.edu.cn/bioinf/plant-multi/).

### Phylogenetic analysis, gene structure, and conserved motifs of StGRFs

To visualize the evolutionary relationship of GRF family members, full-length amino acid sequences of StGRFs, AtGRFs, OsGRFs and PtGRFs were then aligned using ClustalW. A phylogenetic tree was constructed using the neighbor-joining method in MEGA11 software (https://www.megasoftware.net/) and the bootstrap test was carried out with 1000 replicates. A homology analysis of potato GRF proteins was conducted by aligning of the amino acid sequences using ClustalW (https://www.genome.jp/tools-bin/clustalw), and the resulting alignment was visualized using the SnapGene tool. The map of exon-intron structures of the *StGRF* genes were visualized using GSDS2.0 (Gene Structure Display Server 2.0, http://gsds.cbi.pku.edu.cn/index.php). Furthermore, the MEME tool (https://meme-suite.org/meme/tools/meme) was employed to predict conserved motifs of the potato GRF family member. The number of motifs was set to 8 and zero or one occurrence per sequence.

### *Cis*-acting element analysis of the *StGRF* promoters

The 2000 bp upstream sequences of *StGRF* genes were extracted using TBtools software (Chen et al., 2020) based on the full-length DNA sequence of the potato genome. The *cis*-acting elements in the potential promoter regions were identified using the PlantCARE database (http://bioinformatics.psb.ugent.be/webtools/plantcare/html/). The potential interactions of transcription factors in the 2000 bp upstream regions of *StGRF* genes were predicted using the Plant Transcriptional Regulatory Map (http://plantregmap.gao-lab.org/) with the following parameters: the P-value was set to 1e-6, and *Arabidopsis thaliana* as set to as the reference species. The results of the prediction were visualized using Cytoscape software (v3.9.1). The occurrence frequencies of transcription factors were employed to generate a wordcloud using the wordcloud package in the R project.

### Expression profiles of *StGRF* genes

The expression profiles of *StGRF* genes were determined using RNA-Seq data available at the SpudDB (http://spuddb.uga.edu/). The tissue specificity of different tissues (tuber cortex, tuber pith, tuber peel and shoot apex) and organs (leaf, stem, flower, root, stamen, petiole, stolon, mature tuber and young tuber) and potato plants subjected to diverse stresses (microbial pathogen infection, salt, heat, BAP, IAA, ABA and GA3) was analyzed. Gene expression levels of *StGRF*s were represented by reads per kb per million reads (RFKM). The heatmap of the expression patterns was constructed using TBtools (https://github.com/CJ-Chen/TBtools/).

### RNA isolation and RT-qPCR

Total RNA was extracted using the RNA simple Total RNA Kit (Cat. No. DP419, TIANGEN) in accordance with the manufacturer’s instructions. RNA quality was analyzed using a NanoDrop One spectrophotometer and agarose gel electrophoresis. One microgram of total RNA was employed to synthesize the first strand of cDNA using the HiFiScript gDNA Removal cDNA Synthesis Kit (Cat. No. CW2020M, CWBIO) according to the manufacturer’s instructions. The polymerase chain reaction (PCR) was conducted in a total volume of 10 µL, comprising 5 µL of 2×qPCR Smart Mix (SYBR Green) (Cat. No. DY20302, DEEYEE), 0.5 µL (10 µM) of each gene-specific primer, 3.5 µL of ddH_2_O, and 1 µL of cDNA. The target gene was detected using the QuantStudioTM7 Flex System (Applied Biosystems, Thermo Fisher Scientific, USA). *StUBI3* was employed as an internal control, and the primers used for the qPCR are listed in **Supplementary Table S1**. The 2^-ΔΔCT^ method was used to calculate the relative gene expression, and all RT-qPCR experiments were performed in triplicate.

## Results

### Identification of *StGRF* genes in the potato genome

A total of 12 *GRF* family members in potato were identified and named *StGRF1* to *StGRF12* according to their physical locations on the chromosomes (**Table 1**) through the use of bi-directional BLAST and conserved domain analysis. Amino acid length analysis revealed a considerable range in the amino acid lengths of the *StGRF* proteins, with *StGRF8* having the shortest length of 226 amino acids and *StGRF2* having the longest length of 599 amino acids (**Table 1**). The molecular weight of the StGRFs exhibited a similarly wide range, from 26.02 kDa (*StGRF8*) to 64.52 kDa (*StGRF2*), while the isoelectric point of *StGRFs* displayed a similar range, from 6.31 (StGRF3) to 9.20 (StGRF1 and StGRF8) (**Table 1**). The 12 *StGRF* genes were unevenly distributed across nine chromosomes (**Table 1**). Among the 12 *StGRF* genes, the chromosome 8 contains four *StGRF* genes, namely *StGRF6*, *StGRF7*, *StGRF8* and *StGRF9*. In contrast, the remaining chromosomes each contain only one *StGRF* gene (**Table 1**). Subcellular localization prediction indicated that all the StGRF proteins were localized in the nucleus (**Table 1**).

**Table 1:** Members of the *GRF* gene family in the potato genome.

Name	Gene ID	Gene length (bp)	Protein length (aa)	Chromosome location	Molecular weight (kDa)	pI	Prediction of the subcellular localization
StGRF1	Soltu.DM.01G032200	3805	420	1	46.61	9.20	Nucleus
StGRF2	Soltu.DM.02G027350	4632	599	2	64.52	7.28	Nucleus
StGRF3	Soltu.DM.03G021710	2573	469	3	51.53	6.31	Nucleus
StGRF4	Soltu.DM.04G032220	3375	597	4	64.33	8.48	Nucleus
StGRF5	Soltu.DM.07G012510	1818	340	7	39.08	8.88	Nucleus
StGRF6	Soltu.DM.08G003530	5386	352	8	38.59	8.84	Nucleus
StGRF7	Soltu.DM.08G021510	3887	341	8	38.08	8.14	Nucleus
StGRF8	Soltu.DM.08G026330	1644	226	8	26.02	9.20	Nucleus
StGRF9	Soltu.DM.08G030120	1969	422	8	45.99	7.59	Nucleus
StGRF10	Soltu.DM.09G004090	4404	377	9	41.79	8.82	Nucleus
StGRF11	Soltu.DM.10G024900	4273	391	10	42.65	8.59	Nucleus
StGRF12	Soltu.DM.12G004310	4164	352	12	39.31	8.11	Nucleus

### Phylogenetic, conserved motif and gene structure analysis

**Figure 1:**
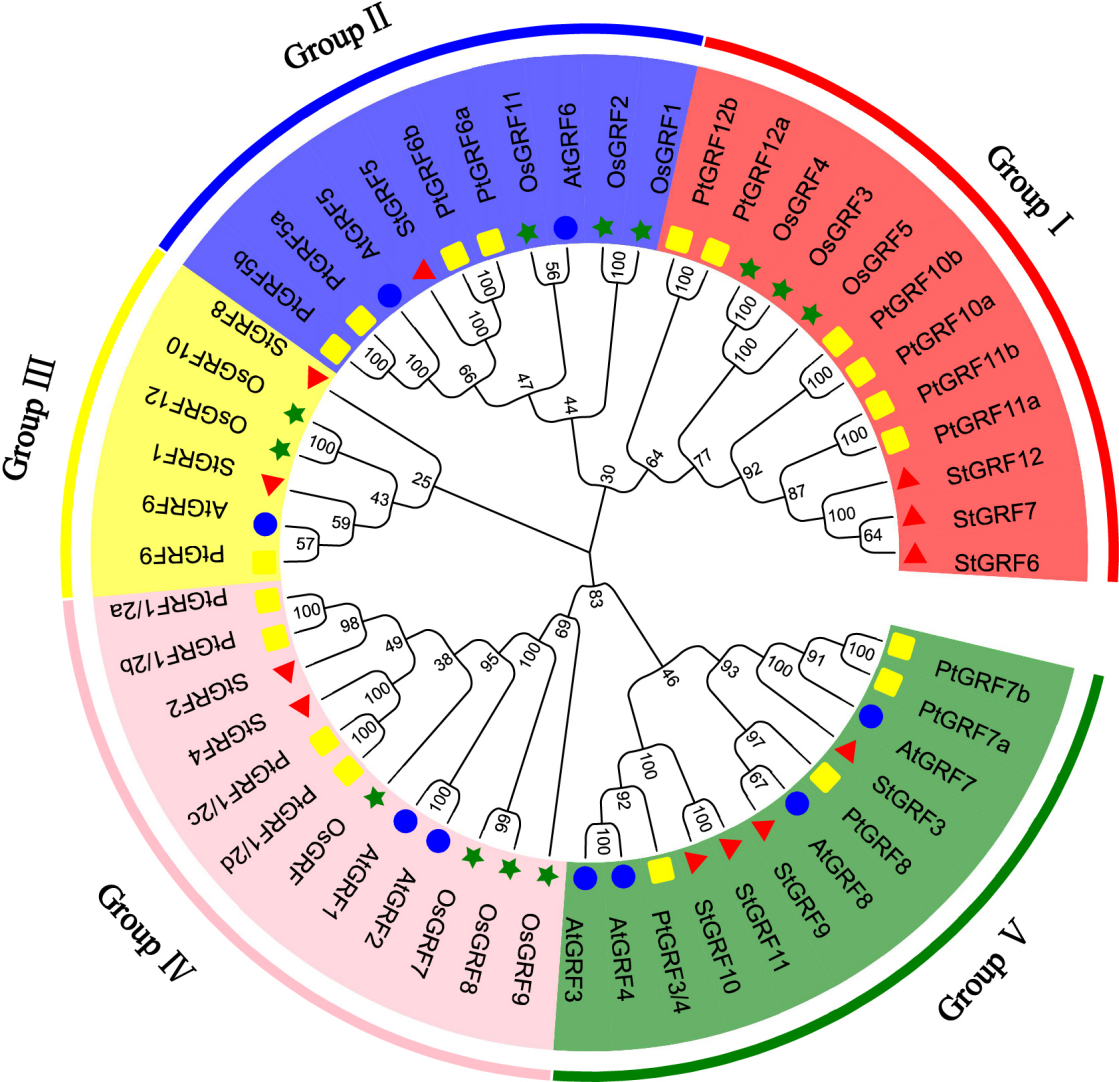
Phylogenetic tree of *GRF* proteins from *Arabidopsis thaliana* (At), *Oryza sativa* (Os), *Solanum tuberosum* (St), and *Populus trichocarpa* (Pt). Blue circle: *Arabidopsis thaliana* (At), green pentagram: *Oryza sativa* (Os), red triangle: *Solanum tuberosum* (St), yellow square: *Populus trichocarpa* (Pt).

**Figure 2:**
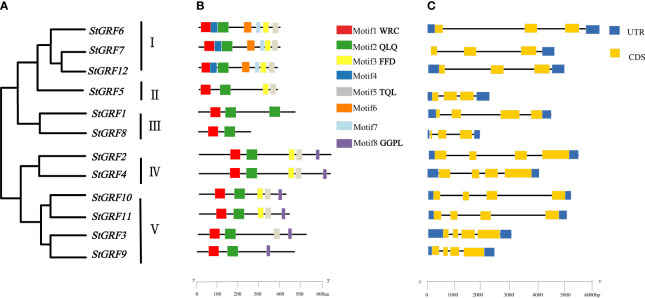
Phylogenetic tree, gene structure and motif of the *GRF* gene family in potato (*Solanum tuberosum*). **(A)** Phylogenetic relationship of *StGRF* proteins. **(B)**The distribution of eight conserved motifs in *StGRF* proteins, identified by the MEME program, as indicated by different colored blocks. **(C)** Exon/intron structures of *StGRFs*. The exons and introns were represented by yellow boxes and black lines, respectively. The dark blue boxes indicate the upstream and downstream untranslated regions, respectively.

To explore the evolutionary relationships and sequence homology among GRF proteins from potato, *Arabidopsis* (Kim et al., 2003), rice (Choi et al., 2004) and poplar (Wang et al., 2020), a neighbor-joining phylogenetic tree was constructed using MEGA11 software (**Figure 1**). The evolutionary relationships of the GRFs were analyzed and a total of 52 GRFs from four plant species were clustered into five subgroups with supported bootstrap values. These were designated as Group I, Group II, Group III, Group IV and Group V (**Figure 1**). A total of three, one, two, two, and four StGRF members were assigned to subgroups I, II, III, IV and V, respectively (**Figure 1**). The phylogenetic tree indicates that the StGRFs are more closely related to PtGRFs and AtGRFs than with to OsGRFs (**Figure 1**). This could be attributed to the fact that potato, poplar and *Arabidopsis* are dicotyledonous plants. In order to ascertain the structural diversity and functional prediction of StGRFs, multiple alignments of the amino acid sequences of StGRF family members were performed (**Figure 2A**). A total of eight conserved motifs were identified, with the length of these motifs ranging from 9 to 41 amino acids (**Supplementary Table S2**). Among these, motifs 1 and 2 were respectively annotated as the WRC and QLQ domain, which were included in all StGRF family members (**Figures 2A, B**), indicating that two domains are highly conserved. All family members, with the exception of *StGRF1*, *StGRF8* and *StGRF9*, contained a TQL (Thr, Gln, Leu) domain at the C-terminus (**Figures 2A, B**). In contrast, the C-termini of all StGRF members, with the exception of *StGRF1*, *StGRF3*, *StGRF8* and *StGRF9*, were found to contain an FFD (Phe, Phe, Asp) domain (**Figures 2A, B**). *StGRF2*, *StGRF3, StGRF4, StGRF9, StGRF10*, and *StGRF11* possess a GGPL (Gly, Gly, Pro, Leu) domain at the C-terminus (**Figures 2A, B**). A multiple sequence alignment of the core QLQ and WRC domain of StGRFs is presented in **Supplementary Figure S1**. It is notable that the characteristics of these motifs were consistent within the same cluster (**Figures 2A, B**). For example, *StGRF6*, *StGRF7* and *StGRF12* exhibited seven common conserved motifs (motifs 1, 2, 3, 4, 5, 6 and 7) (**Figures 2A, B**). The gene structure of the *StGRF* gene family was further analyzed using the GSDS online tool. The *StGRFs* exhibited a length range of 1000 bp to 6000 bp, with the number of introns was found to be 2 to 3 (**Figure 2C**). *StGRFs* from the same clusters exhibited similar exon/intron structure patterns (**Figure 2**), suggesting that phylogenetic relationships among gene family members are highly correlated with gene structure. Furthermore, *StGRF7* has only a 3’ untranslated region (UTR), whereas the other 11 *StGRF* genes possess 5’- and 3’-UTRs at both ends.

### *Cis*-acting elements in the promoter regions of *StGRF* genes

**Figure 3:**
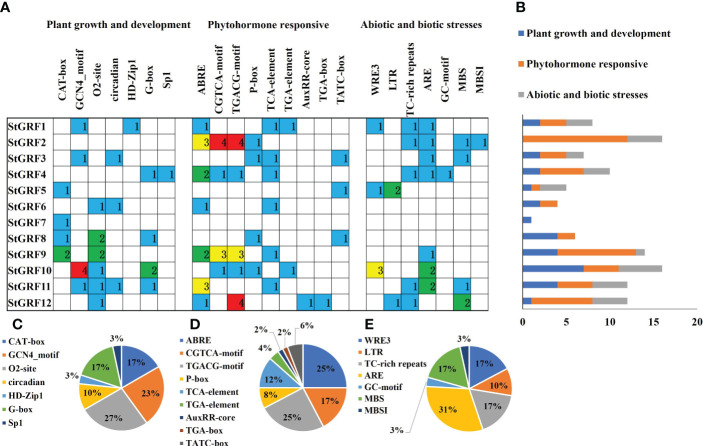
*Cis*-acting element analysis of the potato *GRF* family genes. The different colors represent different *cis*-acting elements. **(A)** Investigation of the number of *cis*-acting elements in the *StGRF* promoter regions. **(B)** Statistics for the number of the promoter elements in three major subfamilies. **(C–E)** The proportion of each promoter in the category.

*Cis*-acting elements within the gene promoters are specific binding sites for proteins involved in the initiation and regulation of gene transcription. To gain further insights into the functions of *cis*-acting elements within the promoter region of *StGRFs*, the promoter sequences for *StGRFs* were submitted to PlantCARE for prediction (**Figure 3**). The *cis*-acting elements within the *StGRFs* promoters are primarily responsible for plant growth and development, as well as responses to hormones and abiotic or biotic stresses (**Figure 3**). *StGRF2*, *StGRF9* and *StGRF12* were found to contain several *cis*-acting elements closely associated with hormone responses, including ABRE (related to the abscisic acid responsiveness), AuxRR-core (auxin responsiveness), TCA-element (involved in salicylic acid responsiveness), CGTCA- and TGACG-motif (involved in MeJA-responsiveness), TATC-box and P-box (gibberellin-responsive element) (**Figure 3**). The majority of *StGRF* genes contained MeJA-responsive elements, CGTCA and TGACG motifs, which were observed 22 times, representing 42% of the hormone-related *cis*-acting elements. ABRE, the abscisic acid responsiveness elements, were observed 13 times, which represented 25% of the hormone-responsive elements of *StGRFs* (**Figure 3**). The promoter regions of nine *StGRFs* contain *cis*-acting elements related to environmental stresses (**Figure 3**). For example, LTR, a low-temperature responsiveness *cis*-acting element identified in *StGRF5* and *StGRF12*. MBS, a drought-inducibility *cis*-acting element identified in *StGRF2*, *StGRF3*, *StGRF11* and StGRF12. The TC-rich repeats and wound-responsive element 3 (WRE 3), which are known to be involved in wounding and pathogen response, have been identified in *StGRF1, StGRF2*, *StGRF4*, *StGRF5*, *StGRF11* and *StGRF12*. The ARE motif, which is essential for the anaerobic induction, has been identified on nine occasions, representing for 31% of the stress-related *cis*-acting elements (**Figure 3**). Furthermore, the promoters of 11 *StGRFs* carry *cis*-acting elements related to plant growth and development (**Figure 3**). The promoters of *StGRF5*, *StGRF7*, *StGRF8*, and *StGRF9* contain the CAT-box, which is associated with meristem development (**Figure 3**). Similarly, the promoters of *StGRF1*, *StGRF3*, *StGRF10*, and *StGRF11* contain the GCN4 motif, which is involved in plant endosperm development (**Figure 3**). The promoters of *StGRF6*, *StGRF8*, *StGRF9*, *StGRF10*, *StGRF11*, and *StGRF12* contain the O2-site, which is involved in the regulation of zein metabolism (**Figure 3**). The O2-site and GCN4 motifs were identified on 8 and 7 occasions, respectively, and accounted for 50% of the plant growth and development-related motifs (**Figure 3**). Additionally, *cis*-acting elements related to circadian control (circadian) and light response elements (G-box and Sp1) were also identified in the promoters of the *StGRFs* (**Figure 3**).

### Bioinformatic analysis of StGRFs-mediated regulatory network

**Figure 4:**
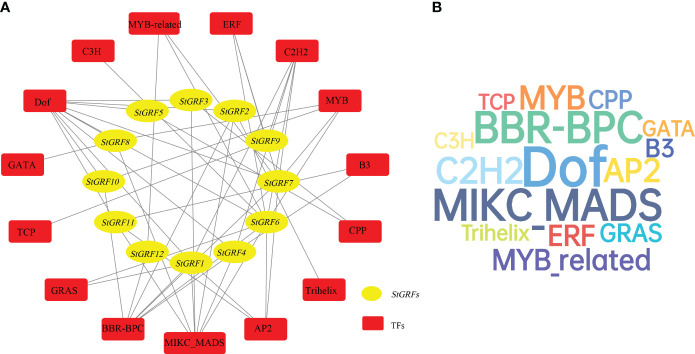
**(A)** The putative transcription factors (TFs) regulatory network analysis of *StGRFs*. Red rounded rectangle nodes represent TFs; yellow ellipse nodes represent *StGRFs*. **(B)** Wordcloud for TFs. The font size is positively correlated with the number of corresponding TFs.

To predict the potential roles of *StGRFs* in potato, a StGRFs-mediated regulatory network was further constructed. Data indicated that a total of 52 transcription factors belonging to 15 different TF families, including Dof, MYB, C2H2, BBR-BPC, MIKC_MADS, AP2, etc., were identified as the potential regulators of *StGRFs* (**Figure 4A**, **Supplementary Table S3**). The predicted TFs were found to be most abundant in the Dof family (54), followed by the MIKC_MADS family (33), the BBR-BPC family (32) and the C2H2 family (20) (**Figure 4B**, **Supplementary Table S3**). In contrast, the least abundant TF families contain only a few members, including the Trihelix family (1), the TCP family (1), the C3H family (1) and the GATA family (2), the CPP family (2) (**Supplementary Table S3**). The predictions indicate that *StGRF6* has the largest class of TFs among all *StGRFs* (10 TFs), followed by *StGRF7* (7 TFs), *StGRF1* (6 TFs) and *StGRF4* (5 TFs) (**Supplementary Table S3**). These enriched transcription factor families may play an essential role in regulating the expression of *StGRFs* in potato. Collectively, the predicted regulatory network of *StGRFs* indicates that they may be involved in a number of biological processes, including plant growth and development, biotic and abiotic stress responses, and network associations.

### Tissue-specific expression of *StGRFs* in potato

**Figure 5:**
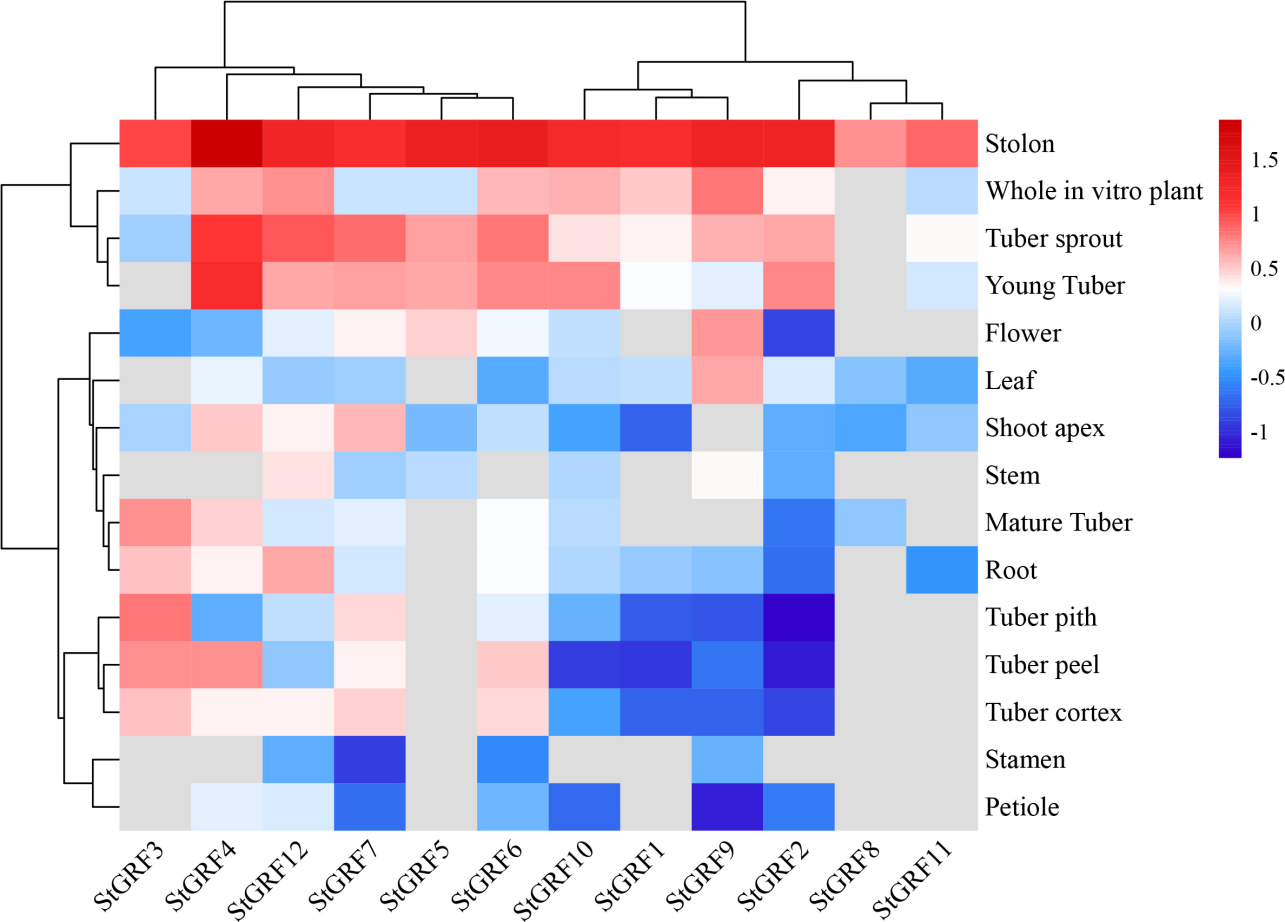
Expression profile analysis of *StGRF* genes in different potato tissues and organs. The color spectrum, ranging from red to blue, represents the expression levels of the genes, with red indicating high expression and blue indicating low expression. Grey indicates the value of zero in the original RNA-Seq data, which the software automatically recognizes this part of the data as ‘missing’.

A comparative analysis of the tissue-specific expression of the 12 *StGRFs* revealed differential expression in different potato tissues and developmental stages (**Figure 5**). The majority of *StGRFs* exhibited low expression in stamens, mature flowers, and mature fruits, whereas high expression was observed in immature tubers, including *StGRF2*, *StGRF4*, *StGRF6*, *StGRF10* and *StGRF12* (**Figure 5**). In heterozygous diploids, all *StGRFs* except for *StGRF1*, *StGRF5, StGRF8* and *StGRF11* were expressed in different organs, including roots, stolons, petioles, flowers and tubers, with varying levels of expression. The expression of different *StGRF* members exhibited tissue-specific characteristics. *StGRF4* exhibited high expression in stolons and young tubers, while lower expression was observed in stems and stamens (**Figure 5**). *StGRF12* was predominantly expressed in roots, stolons, tuber sprouts and young tubers (**Figure 5**). *StGRF9* exhibited high expression in flowers, stolons, and leaves, suggesting an essential role in flower growth and development (**Figure 5**). Notably, *StGRF6* exhibited high expression in four tissues, yet displayed a lower transcript level in stamens, leaves and stems (**Figure 5**). *StGRF3* was highly specifically expressed in stolons, tuber piths and mature tubers, whereas *StGRF7* was highly expressed in stolons and tuber sprouts (**Figure 5**). *StGRF5* showed high expression in stolons, tuber sprouts and young tubers, with very weak expression in other tissues. *StGRF*2 and *StGRF10* were highly expressed in young tubers, tuber sprouts, and stolons, with minimal expression in other tissues. *StGRF1*, *StGRF8*, and *StGRF11* showed weak expression in the majority of the examined tissues, with the exception of stolons (**Figure 5**). Collectively, all the 12 *StGRFs* exhibited distinct expression patterns according to the SpudDB database, indicating that they may have diverse biological functions in various tissues.

### Expression analysis of *StGRF* genes under multiple external stimuli and hormones

**Figure 6:**
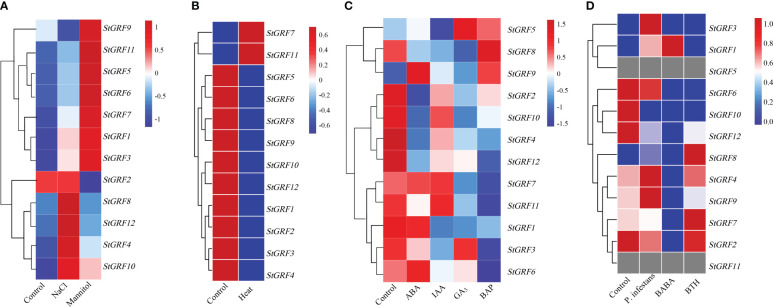
Heatmaps of the expression profiles of ten *StGRF* genes under ten different biotic or abiotic stresses. The transcripts were identified through the use of RNA-Seq technology. **(A)** Salt stresses include 150 mM NaCl for 24 h, and 260 μM mannitol for 24 h. **(B)** The heat stress treatment was 35°C for 24 h. **(C)** The phytohormone treatments included 50 μM ABA (abscisic acid) for 24 h, 10 μM IAA (indole-3-acetic acid) for 24 h, 50 μM GA_3_ (gibberellic acid) for 24 h and 10 μM BAP (6-benzylaminopurine) for 24 h. **(D)** Biotic stresses include *Phytophthora infestans*, the stress elicitors BTH (benzothiadiazole), and BABA (β-aminobutyric acid), which are applied to leaves for 48 hours each. The color patterns, ranging from red (up-regulated expression level) to green (down-regulated expression level), provide an indication of the expression levels of the detected genes under the given conditions. The grey color indicates the value of zero in the original RNA-Seq data, and the software automatically recognizes this part of the data as ‘missing’. The conditions (vertical) and genes (horizontal) with similar profiles were hierarchically clustered (Pearson correlation, average linkage).

The expression patterns of the *StGRF* genes in response to abiotic stresses (salt, heat, osmotic shock) were investigated. Salt stress induced up-regulation of *StGRF1*, *StGRF3*, *StGRF4*, *StGRF5*, *StGRF6*, *StGRF7*, *StGRF8* and *StGRF10*, *StGRF11* and *StGRF12*, and down-regulation of *StGRF9*, whereas *StGRF2* showed negligible changes under the NaCl treatment (**Figure 6A**). A rapid increase in the transcript levels of *StGRF1*, *StGRF3*, *StGRF4*, *StGRF5*, *StGRF6*, *StGRF7*, *StGRF9*, *StGRF10* and *StGRF11* was observed during osmotic shock induced by the mannitol treatment (**Figure 6A**). Upon exposure to heat stress, the mRNA abundance of *StGRF1*, *StGRF2*, *StGRF3*, *StGRF4*, *StGRF5*, *StGRF6*, *StGRF8*, *StGRF9*, *StGRF10* and *StGRF12* was found to be down-regulated, whereas *StGRF7* and *StGRF11* were up-regulated (**Figure 6B**). To ascertain whether *StGRFs* are involved in the hormone response, the expression patterns of *StGRF* genes in potato upon treatment with ABA (abscisic acid), GA_3_ (gibberellic acid), IAA (indole-3-acetic acid) and BAP (6-benzylaminopurine) were investigated (**Figure 6C**). The expression levels of *StGRF6*, *StGRF7*, and *StGRF9* were significantly elevated in response to ABA stimulation, whereas the expression levels of *StGRF2*, *StGRF4*, *StGRF8*, *StGRF10*, *StGRF11* and *StGRF12* were significantly reduced, and *StGRF1* showed no significant changes (**Figure 6C**). Upon IAA treatment, the expression levels of *StGRF1*, *StGRF2*, *StGRF3*, *StGRF4*, *StGRF5*, *StGRF6*, *StGRF8* and *StGRF12* were down-regulated (**Figure 6C**). The application of GA_3_ led to the up-regulation of *StGRF5* and *StGRF9*, while the expression levels of *StGRF1*, *StGRF2*, *StGRF4*, *StGRF6, StGRF7, StGRF8, StGRF10, StGRF11* and *StGRF12* were downregulated (**Figure 6C**). Treatment with BAP induced an increase in *StGRF5*, *StGRF8* and *StGRF9*, while the steady-state mRNA levels of other *StGRF* members exhibited a decrease (**Figure 6C**). Notably, the assays also revealed that the up-regulation of *StGRF1*, *StGRF3*, *StGRF4* and *StGRF9* was observed in leaves infected with the biotic factor *Phytophthora infestans*. Furthermore, except for *StGRF1*, the down-regulation of *StGRF2*, *StGRF4*, *StGRF6*, *StGRF7*, *StGRF9* was observed. The expression of *StGRF10* and *StGRF11* was observed in leaves treated with the chemical elicitor BABA (β-aminobutyric acid), whereas the expression of *StGRF1*, *StGRF6, StGRF9* and *StGRF12* was repressed in leaves treated with the chemical elicitor BTH (benzothiadiazole) (**Figure 6D**). This indicates that *StGRF1* and *StGRF9* play an essential role in potato defense against invading microbes. Taken together, the detailed expression analyses suggest that *StGRF* genes may be involved in the regulation of biotic and abiotic stresses in potato.

### Expression profiles of *StGRFs* during the process of potato tuber dormancy release

**Figure 7:**
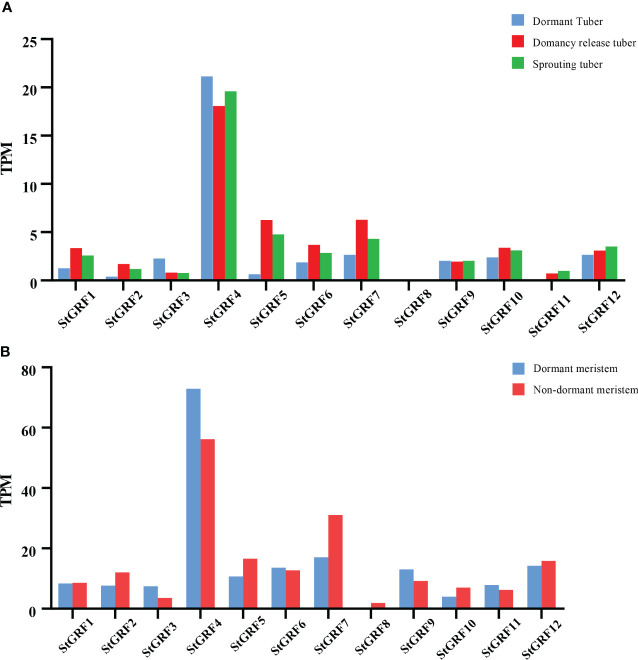
Expression profiles of *StGRFs* during dormancy release. Transcripts from the potato cultivars Longshu3 **(A)**, and Russet Burbank **(B)** were detected using RNA-Seq technology, as described by Liu et al. (Liu et al., 2015) and Campbell et al. (Campbell et al., 2008).

**Figure 8:**
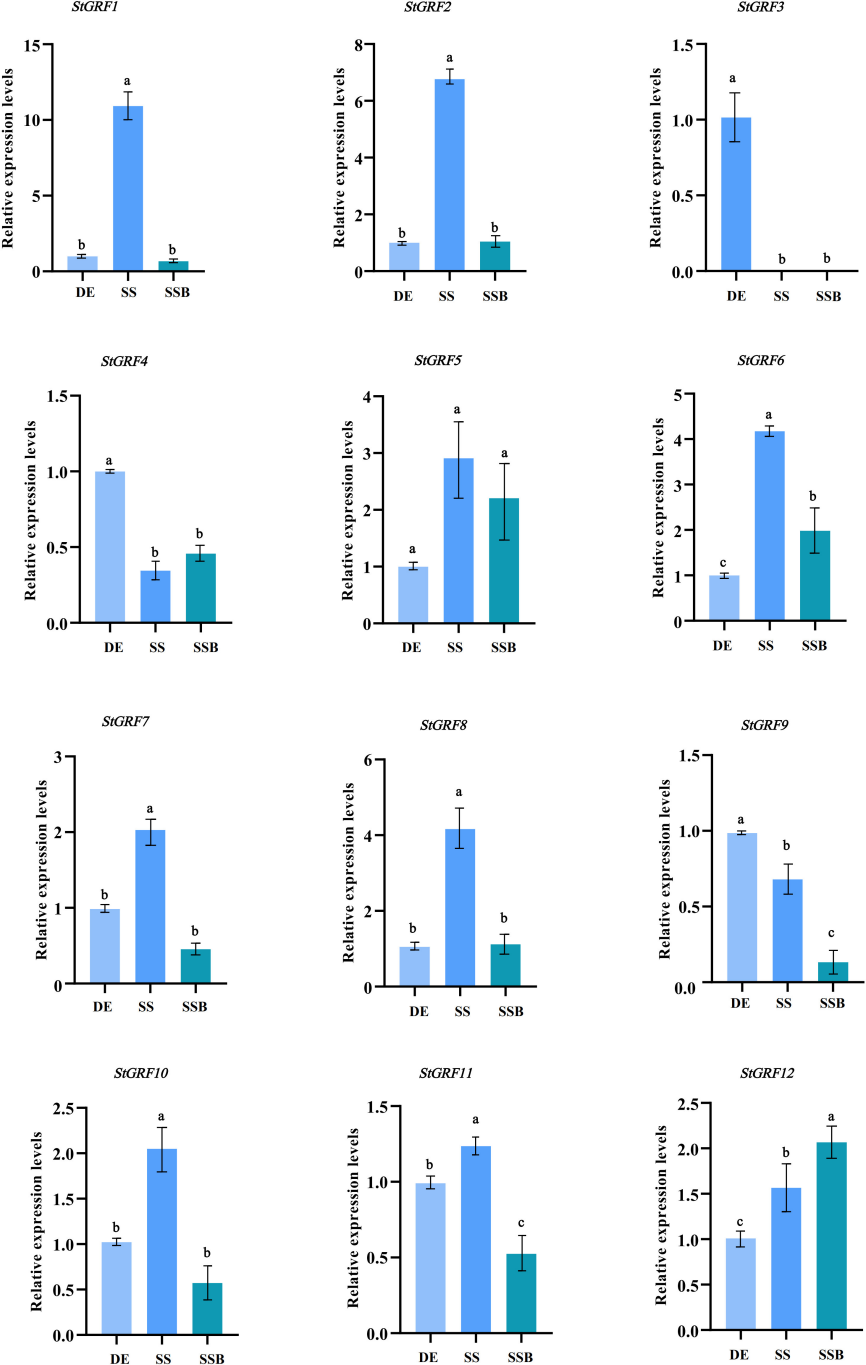
The expression levels of *StGRF* genes from the diploid potato line EB063 during dormancy release were determined by quantitative reverse transcription-polymerase chain reaction (RT-qPCR). Using the dormant eye as a control (DE), data represent the mean ± SEM (n=3). Analysis of variance and multiple comparisons (Tukey) were performed using the software GraphPad Prism9.0. The different letters indicate significant differences between samples (*p* < 0.05), and the same letter indicates that there is no significant difference between samples (*p* ≥ 0.05).

The expression of *StGRF* genes was evaluated based on TPM (transcripts per kilobase of exon model per million mapped reads) values during the process of tuber dormancy release in publicly available RNA-Seq datasets from potato cultivars Longshu3 and Russet Burbank (Spud DB). In the case of the potato cultivar Longshu3, the transcript levels of the *StGRF1*, *StGRF2*, *StGRF5*, *StGRF6*, *StGRF7*, *StGRF10*, *StGRF11* and *StGRF12* increased in the dormancy-release tuber and sprouting tuber compared to the dormant tuber. In contrast, the transcript levels of *StGRF3* and *StGRF4* showed a decrease after tuber dormancy release, while the transcription of *StGRF8* was practically undetectable (**Figure 7A**). In the potato cultivar Russet Burbank, the expression profiles of *StGRF2*, *StGRF5*, *StGRF7*, *StGRF8*, *StGRF10* and *StGRF12* are up-regulated after tuber dormancy release or in nondormant tuber, whereas the expression profiles of *StGRF3*, *StGRF4*, *StGRF6*, *StGRF9* and *StGRF11* decrease after tuber dormancy release or in nondormant tuber (**Figure 7B**). A reduction in the expression of *StGRF3* and *StGRF4* was observed in potato cultivars Longshu3 and Russet Burbank following the release of tuber dormancy. This indicates that these genes may be involved in maintaining tuber dormancy. An up-regulation of *StGRF2*, *StGRF5*, *StGRF7*, *StGRF10* and *StGRF12* was observed in cultivars Longshu3 and Russet Burbank after tuber dormancy release, suggesting an association of these genes with the breaking of tuber dormancy and sprouting. To investigate whether *StGRF* genes were implicated in tuber dormancy, an RT-qPCR assay was conducted on all members of the *StGRF* family to ascertain their respective RNA accumulation profiles during the process of potato tuber dormancy release (**Figure 8**). The mRNA levels of *StGRF5*, *StGRF6*, *StGRF8*, and *StGRF12* were observed to increase after the tuber dormancy release in sprouts, and remained high in apical parts lacking sprouts compared to dormant apical parts. The mRNA levels of *StGRF1*, *StGRF7*, and *StGRF11* were observed to increase following the release of tuber dormancy in sprouts but decreased in apical parts without sprouts. This indicates that these *StGRFs* may be involved in promoting bud outgrowth. The expression of *StGRF4* and *StGRF9* decreased dramatically during tuber dormancy release, indicating that they play a role in of the regulation of tuber sprouting. No transcription was observed for *StGRF3* in sprouts and apical sections. The relative abundance levels of these *StGRFs* as determined by qPCR were found to be generally consistent with those generated from the RNA-Seq datasets (**Figures 7**, **8**), suggesting that *StGRF1*, *StGRF2*, *StGRF5*, *StGRF7*, *StGRF10* and *StGRF12* may be sprout-related genes, while *StGRF4* and *StGRF9* may be dormancy-related genes.

## Discussion

In this study, 12 potato *GRF* genes (*StGRFs*) were identified through database analysis (**Figure 1**), followed by an in-depth investigation of gene structure, protein motifs, phylogenetic relationships, and gene expression patterns. StGRF proteins contain conserved WRC (Motif1) and QLQ (Motif2) domains (**Figure 2**; **Supplementary Figure S1**). The QLQ domain is responsible for protein-protein interactions, whereas the WRC domain is involved in DNA binding and nuclear targeting of the transcription factor (van der Knaap et al., 2000). Notably, StGRF1 contains a second WRC domain in its C-terminal region, similar to BrGRF12 from the Chinese cabbage *Brassica rapa* (Wang et al., 2014). In addition to the N-terminal conserved WRC and QLQ domains, the StGRF proteins carry additional C-terminal motifs, namely FFD, TQL, and GGPL, located in Motif 3, Motif 5, and Motif 8, respectively, in addition to the N-terminal conserved WRC and QLQ domains. The TQL motif, which has been described in AtGRF1 to AtGRF4, as well as in OsGRF1 to OsGRF5 (Omidbakhshfard et al., 2015), is present in StGRF2 to StGRF7, StGRF10 to StGRF12 (**Figure 2**). The FFD motif reported in the sweet orange (*Citrus sinensis*) GRF family members (Fu et al., 2024) is present in StGRF family members StGRF2, StGRF4, StGRF5, StGRF6, StGRF7, StGRF10, StGRF11 and StGRF12 (**Figure 2**). Similarly, the GGPL motif, which was reported in AtGRF1 to AtGRF4, AtGRF7, and AtGRF8 (van der Knaap et al., 2000; Kim et al., 2003; Choi et al., 2004), has been found to be located in StGRF2 to StGRF4, StGRF9 to StGRF11 (**Figure 2**). These TQL, FFD and GGPL motifs are also present in several GRFs from other plant species and are crucial for GRF function in plant tissues and organs (Omidbakhshfard et al., 2015). It is noteworthy that motifs 4, 6 and 7 are exclusive to the StGRF family members StGRF6, StGRF7 and StGRF12, which were categorized as subgroup I (**Figures 1**, **2**). This suggests that these three motifs may be specific to subgroup I. The divergent C-terminal motifs of GRFs act as binding sites for transcriptional co-regulators and also have transcriptional transactivation activities (van der Knaap et al., 2000). Consequently, the conservation of both the N-terminal and C-terminal motifs is of significant importance for the evolutionary expansion and functional conservation of GRF family members in potato.

*GRF* genes are typically highly expressed in relatively active tissues such as germinating seeds and buds (Kim et al., 2003; Horiguchi et al., 2005; Kim et al., 2012; Zhang et al., 2018). RNA-Seq data show that *StGRF* genes are expressed at higher levels in the roots, shoot apices, flowers, and young tubers, where cell proliferation is strong in *Arabidopsis* (Kim et al., 2003; Rodriguez et al., 2010). The increased expression of *StGRF9* in the leaf, flower and stolon, in comparison to the root, suggests a role for this gene in these organs, analogous to its presumed orthologue *AtGRF8* (Kim et al., 2003). *StGRF5* is expressed in the flower (**Figure 5**), in a manner analogous to its putative orthologue *AtGRF5*, which is predominantly expressed in the floral meristem (Pajoro et al., 2014). The overexpression of *AtGRF5* in *Arabidopsis* results in an increase in cell proliferation in leaf primordia, leading to the formation of larger leaves than those observed in wild-type plants (Horiguchi et al., 2005). *StGRF3* is highly expressed in the root and clusters with *AtGRF3*, *AtGRF4* and *AtGRF7* (**Figures 1**, **5**), suggesting that it may perform similar functions in the root. *AtGRF3* is expressed in the meristematic zone and elongation zone of primary and emerging lateral roots (Gorlova et al., 2014; Hepworth and Lenhard, 2014; Lee et al., 2018). *AtGRF4* has been identified in carpels and roots, with the highest levels of expression observed in the root tip and differential zone (Bao et al., 2014). *AtGRF4* is strongly expressed in the meristematic zone but weakly expressed in the elongation zone (Bao et al., 2014). *AtGRF7* is mainly expressed in flowers and shoot tips, with minimal expression in roots (Kim et al., 2003). Furthermore, *AtGRF7* is also expressed in developing tissues, including the vascular tissues, the inflorescence meristem, the bud pistil, the silique replum, the veins of leaf blades, and the petioles of true leaves (Kim et al., 2012). The same subfamily, *StGRF1* and *StGRF8*, demonstrated weak expression in all tissues examined (**Figures 1**, **5**). However, *StGRF6*, *StGRF7* and *StGRF12*, which belong to the same subfamily as their homologs *OsGRF3*, *OsGRF4*, and *OsGRF5*, exhibited high expression in the young tubers, tuber sprouts and stolons (**Figures 1**, **5**). This high expression may be involved in biological pathways that contribute to plant growth and development. The tissue-specific expression profiles of genes are typically associated with *cis*-regulatory elements (Saeed et al., 2006). It was observed that most of the *StGRF*s contain one or more than one *cis*-element related to plant growth and development in their promoter regions (**Figure 3**). The 12 *StGRF* genes containing these *cis*-elements may be involved in a variety of functions (**Figure 3**). The diversity of their functions and the distribution of *cis*-elements in the promoter regions of these genes suggest that *StGRFs* may differentially regulate the expression of genes involved in potato tuber development. Further functional characterization of the *StGRF* genes is essential to gain new insights into the molecular mechanism of tuber development in potato. Tuber initiation and development are of significant importance in potato production. The initiation and development of tubers are regulated by members of the FLOWERING LOCUS T (FT) clade and multiple environmental factors (Navarro et al., 2011). The high expression of the majority of *StGRF* genes in young tubers suggests their involvement in tuber formation and development (**Figure 5**), potentially through the association with other tuber-specific expression genes. For instance, *StGRF4* expression was significantly higher in young tubers than in mature tubers (**Figure 5**), which is consistent with previous findings indicating that *GRFs* are involved in the initial stages of growth and development in various tissues.

A number of studies have investigated the impact of abiotic stresses, including heat, drought, and salinity, on potato yield, tuber quality, and market value (Liu et al., 2009; Liang et al., 2014; Dahal et al., 2019 **;** **Tiwari et al., 2022**). Moreover, it has been demonstrated that *GRFs* may play a role in response to drought and salt stress (Kim et al., 2012). In comparison to the wild-type and *AtGRF7-*overexpressor lines, the *atgrf7* mutant line exhibits increased tolerance to drought and salt stress. Conversely, *AtGRF7* suppresses the expression of osmotic stress-responsive genes, including *DREB2A*, even in the absence of stress treatment (Liu et al., 1998; Kim et al., 2012). This indicates that abiotic stress causes the repression of *AtGRF7* expression, which in turn induces the activation of osmotic stress-responsive genes. It was shown that salt stress resulted in the down-regulation of five *GhGRF* genes (*GhGRF3, GhGRF4, GhGRF5, GhGRF7*, and *GhGRF16*) in *Gossypium hirsutum* (Liu et al., 2022). However, the majority of *StGRF* genes were found to be up-regulated under NaCl stress, with the exception of *StGRF2* and *StGRF9* (**Figure 6A**). Furthermore, the expression levels of *StGRF1*, *StGRF3*, *StGRF5, StGRF6, StGRF7, StGRF9* and *StGRF11* genes showed a significant increase following mannitol treatment (**Figure 6A**). This indicates that osmotic stress is responsible for the activation of *StGRF* gene expression. It was demonstrated that poplar *GRF15* was induced by heat stress. Compared to wild-type poplar plants, the transgenic lines overexpressing *PtGRF15* and lacking the miR396a target sites exhibited enhanced photosynthetic efficiency and heat tolerance (Zhao et al., 2021). The expression of most *StGRF* genes was suppressed under heat stress, while *StGRF7* and *StGRF11* genes exhibited a slight induction (**Figure 6B**). Nevertheless, further research is required to elucidate the underlying mechanisms of these phenomena.

Phytohormones exert a profound influence on the growth, differentiation, and development of plants. Previous reports have shown that GA_3_ treatment increases the expression of several *GRF* genes in rice and cabbage (van der Knaap et al., 2000; Choi et al., 2004; Wang et al., 2014), whereas *AtGRFs* do not appear to be induced by GA_3_ (Kim et al., 2003). KNOTTED1-like homeobox (KNOX) transcription factors are known to restrict cell differentiation and are important regulators of meristem development. GRFs have been demonstrated to act as upstream repressors of *KNOX* genes, which inhibit GA biosynthesis (Kuijt et al., 2014). The application of GA_3_ induces the expression of *KNOX*, which subsequently leads to the up-regulation of *GRFs* at low levels of KNOX expression (Kuijt et al., 2014). The expression of several *NtabGRF* genes in *Nicotiana tabacum* is triggered by a number of different treatments, including GA_3_, IAA, BR, ABA, and BAP (Zhang et al., 2018). Further analysis revealed that the *NtabGRF* promoters contain several hormone-related *cis*-elements, including the GARE-motif, TATC-box, and P-box (gibberellin responsiveness) elements in GA_3_-inducible *NtabGRF* genes and ABRE (abscisic acid responsiveness) elements in ABA-inducible *NtabGRF* genes (Zhang et al., 2018). In *Camellia sinensis*, only one *GRF* gene was induced by GA_3_ treatment, whereas most of *GRFs* were induced by SA or IAA (Wu et al., 2017). This study revealed that the expression levels of *StGRF2*, *StGRF4*, *StGRF8* and *StGRF12* were simultaneously downregulated, while *StGRF9* was induced by GA_3_, IAA, and ABA (**Figure 6C**). The *StGRF12* gene was also differentially downregulated by BAP treatment (**Figure 6C**), suggesting that *StGRF12* may be a key negative regulator of potato in response to GA_3_, IAA, ABA, and BAP treatments. It can be concluded that these *StGRFs* play a crucial role in regulating hormone feedback mechanisms in potato. A promoter analysis revealed that the potential functions of *StGRFs* are induced by different hormones (**Figure 3**). The promoter regions of *StGRF2*, *StGRF3*, *StGRF8* and *StGRF10* contain GA-responsive elements, specifically P-box motifs (**Figure 3**). Similarly, *StGRF12* contains auxin-responsive TGA-box motifs (**Figure 3**). Furthermore, the ABA-responsive ABRE motifs were identified in *StGRF1*, *StGRF2*, *StGRF4*, *StGRF6*, *StGRF9*, *StGRF11*, and *StGRF12* (**Figure 3**). It is therefore postulated that *StGRFs* may have a role in regulating physiological processes related to hormonal feedback mechanisms in potato.

Potato tubers are formed from shortened internodes, which subsequently undergo swelling and the formation of tuber eyes. Meristematic activity in the tuber eyes is completely ceased, resulting in the tuber entering a period of dormancy. Consequently, potato tuber dormancy is confined to the tuber eyes, where the meristem is located, while the remainder of the tuber continues to undergo physiological metabolic activity (Aksenova et al., 2013). It is postulated that changes in meristematic activity represent a pivotal factor in the process of dormancy release. It is hypothesized that the reactivation of meristematic function coincides with the breaking of dormancy. Meristematic activity typically resumes prior to tuber bud emergence, which is referred to as the breaking of dormancy or tuber dormancy release. Subsequently, an increase in cell division results in the visible growth of the bud, which is known as tuber sprouting. This often occurs with buds that are larger than 2 mm. *GRFs* are frequently highly expressed in actively growing tissues. The data indicate that *AtGRFs* are highly expressed in developing and growing tissues where cell proliferation occurs, in different parts of *Arabidopsis* roots and shoots (Kim et al., 2003). Gene expression profiles provide valuable insights into the potential functions of genes. In the present study, publicly available RNA-Seq data and RT-qPCR were used to elucidate the expression of *StGRFs* during potato tuber dormancy and sprouting (**Figures 7**, **8**). The gene expression patterns of *StGRFs* in dormant and sprouting tubers were analyzed in order to identify the complex expression patterns exhibited by these genes (**Figure 8**). The *StGRF* genes displayed spatiotemporally specific patterns (**Figures 7**, **8**). *StGRF2*, *StGRF5*, *StGRF7*, *StGRF10*, and *StGRF12* showed high expression levels following dormancy release, indicating their expression in growing tissues (**Figures 7**, **8**). *AtGRF1*, *AtGRF2*, and *AtGRF3* have been shown to regulate cell proliferation, leaf, and cotyledon development (Kim et al., 2003). *AtGRF5* overexpression results in larger leaf areas due to increased cell number (Horiguchi et al., 2005). Conversely, *ZmGRF10* overexpression in maize leads to a decrease in plant height and leaf size, particularly leaf length, accompanied by impaired cell proliferation (Wu et al., 2014). This indicates that the reduction in leaf size observed in transgenic maize is likely due to a limitation in cell proliferation. Transcriptome data indicated a reduction in the expression level of the *StGRF3* gene following germination, while RT-qPCR results demonstrated that the expression level of the *StGRF3* gene was reduced to zero after germination (**Figures 7**, **8**). This suggests that the *StGRF3* gene may act as a negative regulator in the process of tuber dormancy. The expression of the *StGRF8* gene was not detected during the germination of the potato cultivar Longshu 3. In contrast, in the diploid potato line EB063, *StGRF8* gene expression increased with the release of dormancy, which was consistent with the expression observed in the potato cultivar Russert Burbank (**Figures 7**, **8**). These findings indicate that *StGRF8* exhibits distinct expression patterns during the germination process in different potato genotypes. Therefore, it is therefore necessary to further verify its specific role. Furthermore, *StGRF4* and *StGRF9* exhibited comparable reduced expression levels after the release of dormancy (**Figure 8**), indicating that these two *StGRF* genes may be involved in bud dormancy.

## Conclusions

In this study, 12 members of the potato GRF family were identified and their conserved N-terminal WRC and QLQ domains, as well as C-terminal FFD, TQL, and GGPL motifs were characterized. The intron–exon organization was analyzed, and the evolutionary relationships between the StGRFs and their homologs from two representative plant species were investigated. A total of 30 *cis*-acting elements related to plant growth and development, 30 *cis*-acting elements related to abiotic stress, and 40 *cis*-acting elements related to hormone response were identified in the promoter regions of *StGRFs*. A StGRFs-mediated regulatory network was constructed, comprising 52 transcription factors belonging to 15 different TF families, which were identified as the potential regulators of *StGRFs*. Furthermore, we examined tissue-specific gene expression patterns, as well as gene expression patterns induced by the heat, salt, drought stress, several phytohormones, *Phytophthora infestans*, and chemical elicitors. The involvement of seven *StGRF* genes, *StGRF1*, *StGRF2*, *StGRF5*, *StGRF6*, *StGRF7*, *StGRF10* and *StGRF12*, in tuber sprouting was confirmed. Furthermore, it was demonstrated that two *StGRF* genes, *StGRF4* and *StGRF9*, may be associated with tuber dormancy. The results of our analysis of gene structure, phylogenetic relationships, and transcript expression profiles suggest that *StGRF* genes may have specific roles in potato developmental processes and environmental stresses, particularly during potato tuber dormancy and sprouting.

## Data availability statement

The original contributions presented in the study are included in the article/**Supplementary Material**. Further inquiries can be directed to the corresponding author.

## Author contributions

DC: Writing – original draft. YS: Writing – review & editing. WJ: Writing – original draft. HY: Writing – original draft, Writing – review & editing. SW: Writing – original draft. LY: Supervision, Writing – review & editing. BL: Writing – original draft, Writing – review & editing.

## Supplementary material

Supplementary Figure 1 Multiple sequence alignment **(A)** and composition **(B-C)** of conserved domains (QLQ and WRC domains) in *StGRF* proteins.
