# Supplementary material for: Genome-wide characterization of the GRF transcription factors in potato (Solanum tuberosum L.) and expression analysis of StGRF genes during potato tuber dormancy and sprouting
Source: Front Plant Sci. 2024 Jun 24;15:1417204. doi: 10.3389/fpls.2024.1417204 (PMC11228316; doi:10.3389/fpls.2024.1417204)
Supplement: Supplementary file 1 [file Table_1.docx]

Table S1. Conserved motifs present in the StGRF proteins

| **Motif** | **Width** | **E-value** | **Consensus Sequence** |
| --- | --- | --- | --- |
| Motif 1^1^ | 41 | 3.4e-360 | DPEPGRCRRTDGKKWRCSKDAYPDSKYCERHMHRGRNRSRK |
| Motif 2^2^ | 40 | 4.4e-219 | FTAVQWQELEHQAMIYKYLVAGLPVPPDLVVPIRRSF |
| Motif 3^3^ | 21 | 1.6e-029 | KQEQRSMRPFFDEWPTTKESW |
| Motif 4 | 21 | 4.4e-024 | ISARFFHHPSLGYCSYYGKKF |
| Motif 5^4^ | 18 | 6.4e-021 | STTQLSISIPMAPSDFSS |
| Motif 6 | 26 | 1.2e-014 | YGSTTTKLQMEPASYGINNKEYRYGM |
| Motif 7  Motif 8^5^ | 15  9 | 5.3e-011  5.8e-009 | QHCFFSSDIDSPGTV  GGPLAEVLH |
| Conserved sequences within the ^1^WRC domain; ^2^QLQ motif; ^3^FFD domain; ^4^TQL domain; ^5^GGPL domain. | | | |
