# Supplementary material for: Genome-wide characterization of the GRF transcription factors in potato (Solanum tuberosum L.) and expression analysis of StGRF genes during potato tuber dormancy and sprouting
Source: Front Plant Sci. 2024 Jun 24;15:1417204. doi: 10.3389/fpls.2024.1417204 (PMC11228316; doi:10.3389/fpls.2024.1417204)
Supplement: Supplementary file 2 [file Table_2.docx]

| Table S2. Putative transcription factors (TFs) acting as potential regulators of *StGRFs*. | | | | | | | |
| --- | --- | --- | --- | --- | --- | --- | --- |
| **Pattern name** | **TFs** | **Sequence name** | **Start** | **Stop** | **Strand** | **P-value** | **Matched sequence** |
| AT1G03840 | C2H2 | *StGRF1* | 1488 | 1507 | - | 4.98E-07 | ATTAAAACGACAAAAAAAAG |
| AT1G08810 | MYB | *StGRF6* | 1769 | 1787 | + | 6.03E-07 | TAACCCCAACAACCTCTAC |
| AT1G22810 | ERF | *StGRF9* | 892 | 906 | - | 5.85E-07 | GAGGGTGACGGTGAC |
| AT1G29160 | Dof | *StGRF6* | 1697 | 1717 | - | 2.71E-07 | AGAGAGAAAAAAAAGTGAAGA |
| AT1G34670 | MYB | *StGRF10* | 1969 | 1989 | - | 1.12E-07 | GGGGGGAGGGTTGTTGAAGTT |
| AT1G49480 | B3 | *StGRF4* | 1863 | 1877 | + | 5.36E-07 | GAAAGAAAAAAAAAA |
| AT1G49480 | B3 | *StGRF11* | 878 | 892 | - | 7.48E-07 | AAAAAAAAAAAAAAG |
| AT1G49480 | B3 | *StGRF11* | 879 | 893 | - | 2.02E-07 | AAAAAAAAAAAAAAA |
| AT1G49480 | B3 | *StGRF11* | 880 | 894 | - | 2.02E-07 | AAAAAAAAAAAAAAA |
| AT1G49480 | B3 | *StGRF11* | 881 | 895 | - | 2.02E-07 | AAAAAAAAAAAAAAA |
| AT1G49480 | B3 | *StGRF11* | 882 | 896 | - | 2.02E-07 | AAAAAAAAAAAAAAA |
| AT1G49480 | B3 | *StGRF11* | 883 | 897 | - | 2.02E-07 | AAAAAAAAAAAAAAA |
| AT1G49480 | B3 | *StGRF11* | 884 | 898 | - | 2.02E-07 | AAAAAAAAAAAAAAA |
| AT1G49480 | B3 | *StGRF11* | 885 | 899 | - | 2.02E-07 | AAAAAAAAAAAAAAA |
| AT1G49480 | B3 | *StGRF11* | 886 | 900 | - | 2.02E-07 | AAAAAAAAAAAAAAA |
| AT1G49480 | B3 | *StGRF11* | 887 | 901 | - | 2.02E-07 | AAAAAAAAAAAAAAA |
| AT1G51600 | GATA | *StGRF8* | 467 | 485 | + | 7.13E-07 | CATCATTTTCATCAACAAC |
| AT1G51700 | Dof | *StGRF1* | 1112 | 1132 | + | 3.20E-07 | TAAGAGAAAAAGAAAAAAGAA |
| AT1G51700 | Dof | *StGRF1* | 1119 | 1139 | + | 2.13E-07 | AAAAGAAAAAAGAAAAAAGAA |
| AT1G69120 | MIKC_MADS | *StGRF3* | 1842 | 1853 | - | 8.81E-07 | CTAAAAATGGAA |
| AT1G69570 | Dof | *StGRF1* | 1110 | 1136 | - | 4.12E-08 | TTTTTTCTTTTTTCTTTTTCTCTTATT |
| AT1G69570 | Dof | *StGRF1* | 1117 | 1143 | - | 3.54E-07 | ACTCTTCTTTTTTCTTTTTTCTTTTTC |
| AT1G69570 | Dof | *StGRF1* | 1961 | 1987 | + | 8.42E-07 | GTTTTGCTTTTTTCCTTTTCCTTCCAT |
| AT1G69570 | Dof | *StGRF6* | 1697 | 1723 | + | 7.22E-08 | TCTTCACTTTTTTTTCTCTCTCTGTTC |
| AT1G69570 | Dof | *StGRF7* | 1652 | 1678 | + | 9.74E-07 | ACTTCACTTTATATATTTTCTTTCTTT |
| AT1G71450 | ERF | *StGRF6* | 1797 | 1816 | + | 4.93E-07 | CCCCTCTACCCCCACCCCCA |
| AT1G71450 | ERF | *StGRF7* | 1974 | 1993 | - | 5.84E-07 | CCCCTCCACCCCCACCCCCC |
| AT1G72050 | C2H2 | *StGRF4* | 1046 | 1064 | - | 2.89E-07 | CCTACTCCACCCCCGCCCC |
| AT1G72050 | C2H2 | *StGRF4* | 1049 | 1067 | - | 7.32E-07 | CTCCCTACTCCACCCCCGC |
| AT1G72050 | C2H2 | *StGRF4* | 1052 | 1070 | - | 3.23E-07 | CCCCTCCCTACTCCACCCC |
| AT1G72050 | C2H2 | *StGRF6* | 1799 | 1817 | + | 1.57E-07 | CCTCTACCCCCACCCCCAC |
| AT1G72050 | C2H2 | *StGRF7* | 1976 | 1994 | - | 3.37E-07 | TCCCCTCCACCCCCACCCC |
| AT1G72050 | C2H2 | *StGRF7* | 1979 | 1997 | - | 5.79E-08 | CCCTCCCCTCCACCCCCAC |
| AT1G72050 | C2H2 | *StGRF7* | 829 | 847 | + | 5.41E-08 | CCTCATCCCCCCCCCCCCC |
| AT1G72050 | C2H2 | *StGRF7* | 832 | 850 | + | 3.72E-07 | CATCCCCCCCCCCCCCCCC |
| AT1G72050 | C2H2 | *StGRF7* | 834 | 852 | + | 7.52E-07 | TCCCCCCCCCCCCCCCCCC |
| AT1G72050 | C2H2 | *StGRF7* | 835 | 853 | + | 8.30E-08 | CCCCCCCCCCCCCCCCCCC |
| AT1G72050 | C2H2 | *StGRF7* | 836 | 854 | + | 8.30E-08 | CCCCCCCCCCCCCCCCCCC |
| AT1G72050 | C2H2 | *StGRF7* | 837 | 855 | + | 8.30E-08 | CCCCCCCCCCCCCCCCCCC |
| AT1G72050 | C2H2 | *StGRF7* | 838 | 856 | + | 8.30E-08 | CCCCCCCCCCCCCCCCCCC |
| AT1G72050 | C2H2 | *StGRF7* | 840 | 858 | + | 4.34E-08 | CCCCCCCCCCCCCCCCCAC |
| AT1G72050 | C2H2 | *StGRF9* | 490 | 508 | - | 5.17E-07 | TCTCATCCTCTTCCTCTTG |
| AT1G72050 | C2H2 | *StGRF9* | 493 | 511 | - | 2.10E-07 | TCATCTCATCCTCTTCCTC |
| AT1G72740 | MYB_related | *StGRF6* | 1739 | 1753 | + | 7.05E-08 | CCAAAACCCTAGATC |
| AT1G72740 | MYB_related | *StGRF7* | 1721 | 1735 | + | 4.06E-07 | TCCAAACCCTAGATC |
| AT1G72740 | MYB_related | *StGRF12* | 1816 | 1830 | + | 7.05E-08 | CCAAAACCCTAGATC |
| AT1G74650 | MYB | *StGRF7* | 1974 | 1988 | - | 7.04E-07 | CCACCCCCACCCCCC |
| AT1G77080 | MIKC_MADS | *StGRF2* | 1470 | 1484 | + | 2.15E-07 | TCTTTCTAATTCTGG |
| AT1G79180 | MYB | *StGRF10* | 1978 | 1998 | + | 2.45E-07 | AACCCTCCCCCCCAACCCCAC |
| AT2G01570 | GRAS | *StGRF1* | 1115 | 1134 | + | 4.33E-07 | GAGAAAAAGAAAAAAGAAAA |
| AT2G01570 | GRAS | *StGRF1* | 1471 | 1490 | - | 1.01E-07 | AAGAGAGAGAGAGAGGAAGG |
| AT2G01570 | GRAS | *StGRF6* | 1703 | 1722 | - | 5.80E-07 | AACAGAGAGAGAAAAAAAAG |
| AT2G01930 | BBR-BPC | *StGRF1* | 1465 | 1488 | - | 2.24E-08 | GAGAGAGAGAGAGGAAGGAATATA |
| AT2G01930 | BBR-BPC | *StGRF1* | 1467 | 1490 | - | 1.54E-09 | AAGAGAGAGAGAGAGGAAGGAATA |
| AT2G01930 | BBR-BPC | *StGRF1* | 1469 | 1492 | - | 1.88E-08 | AAAAGAGAGAGAGAGAGGAAGGAA |
| AT2G01930 | BBR-BPC | *StGRF1* | 1471 | 1494 | - | 4.73E-09 | AAAAAAGAGAGAGAGAGAGGAAGG |
| AT2G01930 | BBR-BPC | *StGRF1* | 1473 | 1496 | - | 1.57E-08 | AAAAAAAAGAGAGAGAGAGAGGAA |
| AT2G01930 | BBR-BPC | *StGRF1* | 1475 | 1498 | - | 2.77E-07 | ACAAAAAAAAGAGAGAGAGAGAGG |
| AT2G01930 | BBR-BPC | *StGRF2* | 1834 | 1857 | - | 4.02E-07 | GAGATAGATAGAGAGAGGACAAAA |
| AT2G01930 | BBR-BPC | *StGRF2* | 1836 | 1859 | - | 1.03E-07 | GAGAGATAGATAGAGAGAGGACAA |
| AT2G01930 | BBR-BPC | *StGRF2* | 1838 | 1861 | - | 1.30E-08 | AAGAGAGATAGATAGAGAGAGGAC |
| AT2G01930 | BBR-BPC | *StGRF2* | 1840 | 1863 | - | 6.81E-07 | TTAAGAGAGATAGATAGAGAGAGG |
| AT2G01930 | BBR-BPC | *StGRF2* | 1842 | 1865 | - | 6.31E-07 | CTTTAAGAGAGATAGATAGAGAGA |
| AT2G01930 | BBR-BPC | *StGRF4* | 1909 | 1932 | - | 2.19E-07 | AAGAAACTGAAAGAAAGAGACAGA |
| AT2G01930 | BBR-BPC | *StGRF7* | 1802 | 1825 | - | 2.36E-07 | TATACACAGAGAGAGAGAGAAATG |
| AT2G01930 | BBR-BPC | *StGRF10* | 1854 | 1877 | - | 8.96E-07 | CAGTGAGAGAGAAGGAGGGGATAA |
| AT2G01930 | BBR-BPC | *StGRF12* | 1644 | 1667 | - | 2.07E-07 | GAGTGGGGTTGAAGGAGAAAGAGA |
| AT2G20110 | CPP | *StGRF3* | 411 | 425 | - | 1.67E-07 | TTTTGAATTTTTTAA |
| AT2G28810 | Dof | *StGRF1* | 1117 | 1137 | - | 6.84E-08 | CTTTTTTCTTTTTTCTTTTTC |
| AT2G28810 | Dof | *StGRF3* | 1773 | 1793 | + | 8.32E-07 | TTTTTCTGTTTTGCCTTTTTG |
| AT2G28810 | Dof | *StGRF4* | 384 | 404 | + | 4.45E-07 | TATTTTTTTTTTGACTTTTTT |
| AT2G28810 | Dof | *StGRF6* | 1689 | 1709 | + | 3.51E-07 | TCTTTCTTTCTTCACTTTTTT |
| AT2G28810 | Dof | *StGRF7* | 1191 | 1211 | - | 2.59E-09 | TTTTCTCTTTTTTACTTTTTG |
| AT2G28810 | Dof | *StGRF8* | 1227 | 1247 | - | 3.92E-07 | TTTTTGTTTTTTTTCTTTTTC |
| AT2G37590 | Dof | *StGRF7* | 1191 | 1205 | - | 1.29E-08 | CTTTTTTACTTTTTG |
| AT2G40340 | ERF | *StGRF7* | 1972 | 1992 | + | 4.22E-07 | GTGGGGGGTGGGGGTGGAGGG |
| AT2G45660 | MIKC_MADS | *StGRF1* | 1112 | 1132 | - | 8.20E-07 | TTCTTTTTTCTTTTTCTCTTA |
| AT2G45660 | MIKC_MADS | *StGRF1* | 1114 | 1134 | - | 9.12E-08 | TTTTCTTTTTTCTTTTTCTCT |
| AT2G45660 | MIKC_MADS | *StGRF1* | 1115 | 1135 | - | 6.81E-07 | TTTTTCTTTTTTCTTTTTCTC |
| AT2G45660 | MIKC_MADS | *StGRF1* | 1116 | 1136 | - | 1.15E-08 | TTTTTTCTTTTTTCTTTTTCT |
| AT2G45660 | MIKC_MADS | *StGRF1* | 1119 | 1139 | - | 8.90E-07 | TTCTTTTTTCTTTTTTCTTTT |
| AT2G45660 | MIKC_MADS | *StGRF1* | 1120 | 1140 | - | 5.15E-07 | CTTCTTTTTTCTTTTTTCTTT |
| AT2G45660 | MIKC_MADS | *StGRF1* | 1121 | 1141 | - | 3.17E-07 | TCTTCTTTTTTCTTTTTTCTT |
| AT2G45660 | MIKC_MADS | *StGRF1* | 1122 | 1142 | - | 3.91E-07 | CTCTTCTTTTTTCTTTTTTCT |
| AT2G45660 | MIKC_MADS | *StGRF1* | 1469 | 1489 | + | 1.49E-08 | TTCCTTCCTCTCTCTCTCTCT |
| AT2G45660 | MIKC_MADS | *StGRF1* | 1471 | 1491 | + | 4.30E-08 | CCTTCCTCTCTCTCTCTCTTT |
| AT2G45660 | MIKC_MADS | *StGRF1* | 1473 | 1493 | + | 3.45E-09 | TTCCTCTCTCTCTCTCTTTTT |
| AT2G45660 | MIKC_MADS | *StGRF1* | 1475 | 1495 | + | 3.45E-09 | CCTCTCTCTCTCTCTTTTTTT |
| AT2G45660 | MIKC_MADS | *StGRF1* | 1477 | 1497 | + | 3.84E-08 | TCTCTCTCTCTCTTTTTTTTG |
| AT2G45660 | MIKC_MADS | *StGRF1* | 1815 | 1835 | + | 3.56E-07 | CTTTTTTGTTTTCTTCTCTCT |
| AT2G45660 | MIKC_MADS | *StGRF1* | 1962 | 1982 | + | 2.35E-07 | TTTTGCTTTTTTCCTTTTCCT |
| AT2G45660 | MIKC_MADS | *StGRF3* | 1472 | 1492 | - | 6.03E-07 | TTCTCTCCTCTCTCTCTCCTT |
| AT2G45660 | MIKC_MADS | *StGRF4* | 1858 | 1878 | - | 1.41E-07 | GTTTTTTTTTTCTTTCTTTTG |
| AT2G45660 | MIKC_MADS | *StGRF4* | 96 | 116 | - | 4.62E-07 | CCTTTTCTTCTTTCTATTTTT |
| AT2G45660 | MIKC_MADS | *StGRF6* | 1703 | 1723 | + | 1.80E-07 | CTTTTTTTTCTCTCTCTGTTC |
| AT2G45660 | MIKC_MADS | *StGRF8* | 1226 | 1246 | - | 2.44E-08 | TTTTGTTTTTTTTCTTTTTCT |
| AT2G45660 | MIKC_MADS | *StGRF11* | 875 | 895 | + | 6.17E-08 | TGTCTTTTTTTTTTTTTTTTT |
| AT2G45660 | MIKC_MADS | *StGRF11* | 876 | 896 | + | 7.05E-07 | GTCTTTTTTTTTTTTTTTTTT |
| AT2G45660 | MIKC_MADS | *StGRF11* | 877 | 897 | + | 5.42E-08 | TCTTTTTTTTTTTTTTTTTTT |
| AT2G45660 | MIKC_MADS | *StGRF11* | 878 | 898 | + | 6.17E-08 | CTTTTTTTTTTTTTTTTTTTT |
| AT2G45660 | MIKC_MADS | *StGRF11* | 879 | 899 | + | 4.18E-08 | TTTTTTTTTTTTTTTTTTTTT |
| AT2G45660 | MIKC_MADS | *StGRF11* | 880 | 900 | + | 4.18E-08 | TTTTTTTTTTTTTTTTTTTTT |
| AT2G45660 | MIKC_MADS | *StGRF11* | 881 | 901 | + | 4.18E-08 | TTTTTTTTTTTTTTTTTTTTT |
| AT2G45660 | MIKC_MADS | *StGRF11* | 882 | 902 | + | 5.06E-07 | TTTTTTTTTTTTTTTTTTTTA |
| AT3G06740 | GATA | *StGRF8* | 467 | 485 | + | 9.02E-07 | CATCATTTTCATCAACAAC |
| AT3G10030 | Trihelix | *StGRF2* | 372 | 385 | + | 1.94E-07 | AAGTAACGGCGTTA |
| AT3G12130 | C3H | *StGRF6* | 1963 | 1976 | + | 6.55E-09 | AAGCAAAAAGGTGA |
| AT3G13810 | C2H2 | *StGRF1* | 1486 | 1506 | + | 2.82E-07 | CTCTTTTTTTTGTCGTTTTAA |
| AT3G22780 | CPP | *StGRF5* | 336 | 350 | - | 4.33E-07 | AATTAAAAATTTAAA |
| AT3G45610 | Dof | *StGRF1* | 1963 | 1981 | + | 3.57E-07 | TTTGCTTTTTTCCTTTTCC |
| AT3G47500 | Dof | *StGRF6* | 1697 | 1715 | - | 6.07E-07 | AGAGAAAAAAAAGTGAAGA |
| AT3G50410 | Dof | *StGRF1* | 1118 | 1138 | - | 2.41E-07 | TCTTTTTTCTTTTTTCTTTTT |
| AT3G50410 | Dof | *StGRF5* | 1332 | 1352 | - | 2.60E-07 | CCCCCTTACCTTTTACTTTTC |
| AT3G50410 | Dof | *StGRF6* | 1688 | 1708 | + | 4.40E-07 | TTCTTTCTTTCTTCACTTTTT |
| AT3G50410 | Dof | *StGRF7* | 1192 | 1212 | - | 1.03E-07 | TTTTTCTCTTTTTTACTTTTT |
| AT3G54340 | MIKC_MADS | *StGRF6* | 737 | 751 | + | 7.04E-07 | TACTAAAAGTGGAAA |
| AT3G55370 | Dof | *StGRF1* | 1114 | 1134 | - | 9.74E-09 | TTTTCTTTTTTCTTTTTCTCT |
| AT3G55370 | Dof | *StGRF1* | 1121 | 1141 | - | 3.06E-08 | TCTTCTTTTTTCTTTTTTCTT |
| AT3G55370 | Dof | *StGRF1* | 1963 | 1983 | + | 1.56E-08 | TTTGCTTTTTTCCTTTTCCTT |
| AT3G55370 | Dof | *StGRF6* | 1699 | 1719 | + | 1.99E-07 | TTCACTTTTTTTTCTCTCTCT |
| AT3G55370 | Dof | *StGRF11* | 874 | 894 | + | 1.53E-07 | CTGTCTTTTTTTTTTTTTTTT |
| AT4G18960 | MIKC_MADS | *StGRF6* | 736 | 754 | - | 2.27E-07 | TTCTTTCCACTTTTAGTAT |
| AT4G21030 | Dof | *StGRF3* | 1779 | 1797 | - | 2.99E-07 | CTAGCAAAAAGGCAAAACA |
| AT4G21040 | Dof | *StGRF10* | 1934 | 1947 | + | 5.72E-07 | AAAAGCTTCTTTTT |
| AT4G27950 | ERF | *StGRF7* | 1974 | 1994 | - | 2.75E-07 | TCCCCTCCACCCCCACCCCCC |
| AT4G38000 | Dof | *StGRF1* | 1117 | 1144 | - | 2.57E-09 | CACTCTTCTTTTTTCTTTTTTCTTTTTC |
| AT4G38000 | Dof | *StGRF1* | 1124 | 1151 | - | 6.33E-07 | CTTGTATCACTCTTCTTTTTTCTTTTTT |
| AT4G38000 | Dof | *StGRF2* | 335 | 362 | - | 2.29E-07 | ATTTTTAAATTTTATTCTTTTCTTTTTT |
| AT4G38000 | Dof | *StGRF6* | 577 | 604 | + | 9.95E-07 | TCTTTTATCTTTTTTGAATTGCTTTTTG |
| AT4G38000 | Dof | *StGRF7* | 1191 | 1218 | - | 3.33E-08 | GCTTGTTTTTTCTCTTTTTTACTTTTTG |
| AT4G38000 | Dof | *StGRF8* | 1227 | 1254 | - | 5.80E-08 | GCTTTATTTTTTGTTTTTTTTCTTTTTC |
| AT4G38000 | Dof | *StGRF11* | 874 | 901 | + | 1.06E-07 | CTGTCTTTTTTTTTTTTTTTTTTTTTTT |
| AT4G38000 | Dof | *StGRF11* | 875 | 902 | + | 5.13E-07 | TGTCTTTTTTTTTTTTTTTTTTTTTTTA |
| AT4G38000 | Dof | *StGRF11* | 877 | 904 | + | 1.35E-07 | TCTTTTTTTTTTTTTTTTTTTTTTTATC |
| AT4G38910 | BBR-BPC | *StGRF1* | 1462 | 1491 | - | 5.04E-07 | AAAGAGAGAGAGAGAGGAAGGAATATAATT |
| AT4G38910 | BBR-BPC | *StGRF1* | 1464 | 1493 | - | 3.05E-08 | AAAAAGAGAGAGAGAGAGGAAGGAATATAA |
| AT4G38910 | BBR-BPC | *StGRF1* | 1466 | 1495 | - | 1.99E-08 | AAAAAAAGAGAGAGAGAGAGGAAGGAATAT |
| AT4G38910 | BBR-BPC | *StGRF1* | 1470 | 1499 | - | 5.48E-07 | GACAAAAAAAAGAGAGAGAGAGAGGAAGGA |
| AT4G38910 | BBR-BPC | *StGRF2* | 1831 | 1860 | - | 4.24E-07 | AGAGAGATAGATAGAGAGAGGACAAAAGGA |
| AT4G38910 | BBR-BPC | *StGRF6* | 1690 | 1719 | - | 6.49E-07 | AGAGAGAGAAAAAAAAGTGAAGAAAGAAAG |
| AT5G02460 | Dof | *StGRF1* | 1117 | 1137 | + | 4.46E-10 | GAAAAAGAAAAAAGAAAAAAG |
| AT5G02460 | Dof | *StGRF1* | 1124 | 1144 | + | 8.79E-07 | AAAAAAGAAAAAAGAAGAGTG |
| AT5G02460 | Dof | *StGRF6* | 1689 | 1709 | - | 7.30E-08 | AAAAAAGTGAAGAAAGAAAGA |
| AT5G02460 | Dof | *StGRF7* | 1191 | 1211 | + | 3.69E-08 | CAAAAAGTAAAAAAGAGAAAA |
| AT5G02460 | Dof | *StGRF8* | 1227 | 1247 | + | 3.54E-07 | GAAAAAGAAAAAAAACAAAAA |
| AT5G03150 | C2H2 | *StGRF1* | 1488 | 1507 | + | 7.20E-07 | CTTTTTTTTGTCGTTTTAAT |
| AT5G05790 | MYB | *StGRF8* | 1386 | 1400 | - | 4.84E-07 | AACCTTATCATAATC |
| AT5G08070 | TCP | *StGRF9* | 578 | 588 | + | 8.65E-07 | TTGGTCCCCAC |
| AT5G17430 | AP2 | *StGRF1* | 1114 | 1133 | + | 1.17E-07 | AGAGAAAAAGAAAAAAGAAA |
| AT5G17430 | AP2 | *StGRF1* | 1121 | 1140 | + | 3.76E-08 | AAGAAAAAAGAAAAAAGAAG |
| AT5G17430 | AP2 | *StGRF1* | 1475 | 1494 | - | 9.57E-10 | AAAAAAGAGAGAGAGAGAGG |
| AT5G17430 | AP2 | *StGRF1* | 1477 | 1496 | - | 1.07E-09 | AAAAAAAAGAGAGAGAGAGA |
| AT5G17430 | AP2 | *StGRF1* | 1479 | 1498 | - | 2.75E-07 | ACAAAAAAAAGAGAGAGAGA |
| AT5G17430 | AP2 | *StGRF6* | 1279 | 1298 | - | 3.81E-08 | AAAGAGGAGGAAGAGAGAAA |
| AT5G17430 | AP2 | *StGRF7* | 1191 | 1210 | + | 3.79E-07 | CAAAAAGTAAAAAAGAGAAA |
| AT5G17430 | AP2 | *StGRF11* | 875 | 894 | - | 5.29E-08 | AAAAAAAAAAAAAAAAGACA |
| AT5G17430 | AP2 | *StGRF11* | 877 | 896 | - | 2.21E-07 | AAAAAAAAAAAAAAAAAAGA |
| AT5G17430 | AP2 | *StGRF11* | 878 | 897 | - | 3.04E-07 | AAAAAAAAAAAAAAAAAAAG |
| AT5G17430 | AP2 | *StGRF11* | 879 | 898 | - | 2.45E-07 | AAAAAAAAAAAAAAAAAAAA |
| AT5G17430 | AP2 | *StGRF11* | 880 | 899 | - | 2.45E-07 | AAAAAAAAAAAAAAAAAAAA |
| AT5G17430 | AP2 | *StGRF11* | 881 | 900 | - | 2.45E-07 | AAAAAAAAAAAAAAAAAAAA |
| AT5G17430 | AP2 | *StGRF11* | 882 | 901 | - | 2.45E-07 | AAAAAAAAAAAAAAAAAAAA |
| AT5G25390 | ERF | *StGRF7* | 1975 | 1995 | - | 4.72E-07 | CTCCCCTCCACCCCCACCCCC |
| AT5G40330 | MYB | *StGRF6* | 1774 | 1784 | - | 9.01E-08 | GAGGTTGTTGG |
| AT5G42520 | BBR-BPC | *StGRF1* | 1466 | 1486 | + | 7.49E-07 | ATATTCCTTCCTCTCTCTCTC |
| AT5G42520 | BBR-BPC | *StGRF1* | 1468 | 1488 | + | 2.48E-08 | ATTCCTTCCTCTCTCTCTCTC |
| AT5G42520 | BBR-BPC | *StGRF1* | 1470 | 1490 | + | 6.61E-09 | TCCTTCCTCTCTCTCTCTCTT |
| AT5G42520 | BBR-BPC | *StGRF1* | 1472 | 1492 | + | 8.92E-09 | CTTCCTCTCTCTCTCTCTTTT |
| AT5G42520 | BBR-BPC | *StGRF1* | 1474 | 1494 | + | 2.54E-08 | TCCTCTCTCTCTCTCTTTTTT |
| AT5G42520 | BBR-BPC | *StGRF1* | 1476 | 1496 | + | 2.99E-08 | CTCTCTCTCTCTCTTTTTTTT |
| AT5G42520 | BBR-BPC | *StGRF2* | 1833 | 1853 | + | 6.32E-07 | CTTTTGTCCTCTCTCTATCTA |
| AT5G42520 | BBR-BPC | *StGRF2* | 1839 | 1859 | + | 7.31E-09 | TCCTCTCTCTATCTATCTCTC |
| AT5G42520 | BBR-BPC | *StGRF2* | 1841 | 1861 | + | 1.65E-08 | CTCTCTCTATCTATCTCTCTT |
| AT5G42520 | BBR-BPC | *StGRF7* | 1799 | 1819 | + | 7.06E-07 | ACACATTTCTCTCTCTCTCTG |
| AT5G42520 | BBR-BPC | *StGRF10* | 1857 | 1877 | + | 9.38E-07 | TCCCCTCCTTCTCTCTCACTG |
| AT5G42520 | BBR-BPC | *StGRF12* | 1856 | 1876 | + | 9.98E-07 | AACTTTTGCTCTCTATATCTC |
| AT5G60850 | Dof | *StGRF7* | 1191 | 1204 | + | 5.60E-07 | CAAAAAGTAAAAAA |
| AT5G62470 | MYB | *StGRF7* | 1971 | 1988 | + | 4.51E-07 | AGTGGGGGGTGGGGGTGG |
| AT5G62940 | Dof | *StGRF1* | 1109 | 1129 | + | 4.20E-07 | GAATAAGAGAAAAAGAAAAAA |
| AT5G62940 | Dof | *StGRF1* | 1116 | 1136 | + | 2.08E-07 | AGAAAAAGAAAAAAGAAAAAA |
| AT5G62940 | Dof | *StGRF6* | 1697 | 1717 | - | 6.91E-07 | AGAGAGAAAAAAAAGTGAAGA |
| AT5G62940 | Dof | *StGRF7* | 1875 | 1895 | + | 9.35E-07 | AGTGAAGTGAAAAAGGGGAAA |
| AT5G66730 | C2H2 | *StGRF1* | 1491 | 1507 | - | 8.14E-07 | ATTAAAACGACAAAAAA |
| AT5G66940 | Dof | *StGRF1* | 1111 | 1139 | - | 8.45E-10 | TTCTTTTTTCTTTTTTCTTTTTCTCTTAT |
| AT5G66940 | Dof | *StGRF1* | 1118 | 1146 | - | 1.94E-08 | ATCACTCTTCTTTTTTCTTTTTTCTTTTT |
| AT5G66940 | Dof | *StGRF4* | 1851 | 1879 | - | 6.29E-07 | TGTTTTTTTTTTCTTTCTTTTGTTGTTTT |
| AT5G66940 | Dof | *StGRF4* | 382 | 410 | + | 3.24E-07 | CATATTTTTTTTTTGACTTTTTTTTTGTT |
| AT5G66940 | Dof | *StGRF6* | 1687 | 1715 | + | 3.64E-08 | CTTCTTTCTTTCTTCACTTTTTTTTCTCT |
| AT5G66940 | Dof | *StGRF7* | 1185 | 1213 | - | 2.77E-09 | TTTTTTCTCTTTTTTACTTTTTGAAATTT |
| AT5G66940 | Dof | *StGRF8* | 1221 | 1249 | - | 3.61E-07 | ATTTTTTGTTTTTTTTCTTTTTCTACTTG |
