# Supplementary material for: Genome-wide characterization of the GRF transcription factors in potato (Solanum tuberosum L.) and expression analysis of StGRF genes during potato tuber dormancy and sprouting
Source: Front Plant Sci. 2024 Jun 24;15:1417204. doi: 10.3389/fpls.2024.1417204 (PMC11228316; doi:10.3389/fpls.2024.1417204)
Supplement: Supplementary file 3 [file Table_3.docx]

Table S3. Primers used in this study

| **Primer name** | **Oligonucleotide sequence (5’→3’)** | **bp** | **Tm** |
| --- | --- | --- | --- |
| GRF1-F | CATCTATTCTCTGCCACCCCTC | 22 | 58.44 |
| GRF1-R | GAAACTGAGCTGCTGTGAACAG | 22 | 59.11 |
| GRF2-F | TTTGGATCATCTCCCACAGGTG | 22 | 57.10 |
| GRF2-R | CGTCATCACACATGGCAATACC | 22 | 57.15 |
| GRF3-F | CCGTTCAACAAAGCAGCCTC | 20 | 58.02 |
| GRF3-R | ATCCAGCCCGAAGTTCTGTG | 20 | 57.79 |
| GRF4-F | TCATGGGAATCTTCCTTGGGTG | 22 | 57.03 |
| GRF4-R | TGTAAGACTCCGGTTGGTGATG | 22 | 57.26 |
| GRF5-F | CATCATTCACCAACCACACCAC | 22 | 55.69 |
| GRF5-R | TCCATCTGTTCTTCTGCACCTC | 22 | 58.17 |
| GRF6-F | TGTGAGAGGACTAGGAGGTACG | 22 | 58.62 |
| GRF6-R | GATTCGATCACAGGCTCAAACG | 22 | 58.87 |
| GRF7-F | TTAACGAATGGCCCACTGCT | 20 | 57.22 |
| GRF7-R | CATTTGGGGAGCAAGCACTC | 20 | 57.27 |
| GRF8-F | GATGGAACCAGAGCCAAGAAGA | 22 | 58.09 |
| GRF8-R | CTCCACACACTTTCTTGAACGC | 22 | 58.12 |
| GRF9-F | CCACCTACTCCCTACTAACCCA | 22 | 56.88 |
| GRF9-R | TGTTCTCTTACACCTCCATGGC | 22 | 57.29 |
| GRF10-F | AAATCCCTCGTCTGACATCTCG | 22 | 58.78 |
| GRF10-R | ATCGATGACCTTAAAGCCTCGG | 22 | 59.24 |
| GRF11-F | GATGCAGTCAGGGTACTGGG | 20 | 56.82 |
| GRF11-R | GCCACTACATCCCTTGAGCA | 20 | 57.19 |
| GRF12-F | AGTGGCAGGAATTGGAGCAT | 20 | 57.17 |
| GRF12-R | TGGGTTCAAAGCTACGTCGT | 20 | 56.81 |
