# Supplementary figures and images for: Genome-wide characterization of the GRF transcription factors in potato (Solanum tuberosum L.) and expression analysis of StGRF genes during potato tuber dormancy and sprouting
Source: Front Plant Sci. 2024 Jun 24;15:1417204. doi: 10.3389/fpls.2024.1417204 (PMC11228316; doi:10.3389/fpls.2024.1417204)

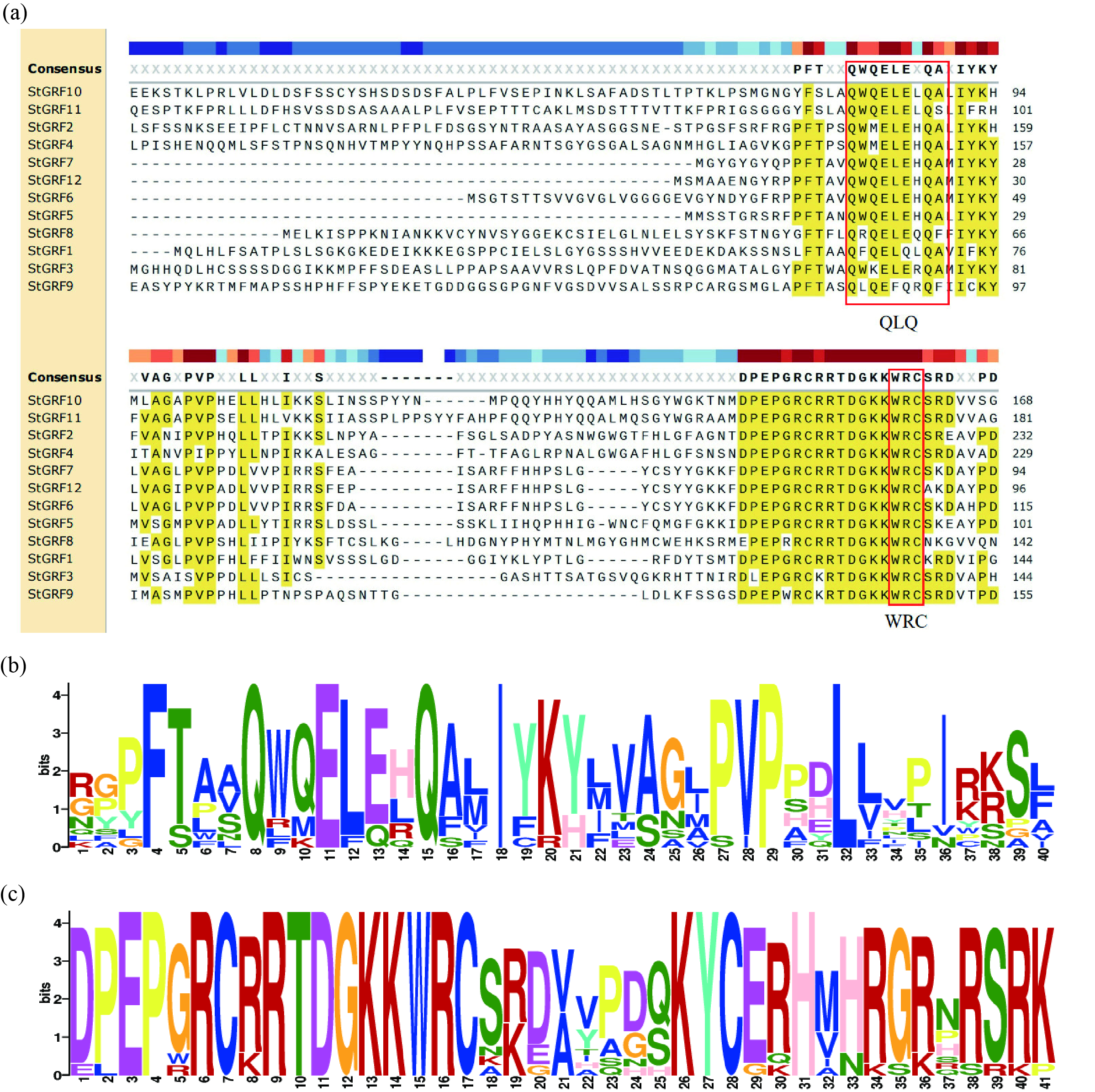

Supplement: Supplementary Figure 1 — Multiple sequence alignment (A) and composition (B-C) of conserved domains (QLQ and WRC domains) in StGRF proteins. [file Image_1.jpeg]
